# How do domain diversity and healthcare system assumptions shape implementation research? A systematic review of theories, models, and frameworks for health digitalization

**DOI:** 10.3389/fdgth.2026.1812300

**Published:** 2026-07-16

**Authors:** Carla Y. Molenaar, Martine C. de Bruijne, Marijke Melles, George Burchell, Hanneke Merten

**Affiliations:** 1 Department of Public and Occupational Health, Amsterdam UMC, Amsterdam, Netherlands; 2 Department of Human Design, Delft University of Technology, Delft, Netherlands; 3 Medical Library, Vrije Universiteit Amsterdam, Amsterdam, Netherlands

**Keywords:** conceptual frameworks, digital health implementation, digital health innovation, health digitalization, healthcare assumptions, models, theories

## Abstract

**Introduction:**

Health digitalization entails a complex array of actors and roles, including healthcare stakeholders and commercial digital-health innovators. The implementation of digital health innovations (DHIs) is notoriously prone to failure. Consequently, many theories, models, and frameworks (TMFs) have been developed to explain or guide the process of health digitalization. Depending on their scholarly origins, these TMFs differ in their perspective, origin, and terminology, leading to knowledge fragmentation and confusion. The aim of this review was to create an overview of the available TMFs, bringing together and clarifying different perspectives of healthcare stakeholders and innovators during the DHI life cycle.

**Methodology:**

This study focused on TMFs describing, explaining, predicting, or prescribing parts of the innovation pathway considering influential factors and actors. Three databases (PubMed, EMBASE, and Web of Science) were searched using the aid of an information specialist in February 2025. We identified relevant keywords for the search by extensively reviewing eHealth and health digitalization literature. Studies were eligible for inclusion if they were published after 2000, written in English, and presented a new or significantly adapted TMF empirically tested in healthcare settings involving a commercial DHI. Studies applying only a known TMF without contextual adaptation or a DHI in the prototype phase were excluded.

**Results:**

From the 4628 potentially relevant articles, 12 fulfilled the inclusion criteria. The included TMFs came from diverse scholarly disciplines, pointing to the multidisciplinary knowledge needed for health digitalization. Four TMFs focused on behavior change of end users, two TMFs explored organizational adoption of the DHI, and six TMFs dealt with factors that shape innovation design and development. Several constructs were found in two or all of the TMF groups. The actors involved came from diverse backgrounds and differed according to the type of DHI and scope of implementation.

**Conclusion:**

The included TMFs synthesized foundational theories and constructs from specific problem areas in health digitalization. Analyzing the reasons for adding certain constructs indicated how actors and researchers in the different groups perceive the landscape of health digitalization and the realities that shape the digitalization process, helping to create a holistic view aiding continuity and outward growth of the digitalization team.

**Systematic Review Registration:**

https://www.crd.york.ac.uk/PROSPERO/view/CRD420251027510, PROSPERO CRD420251027510.

## 1 Introduction

Many scholars have theorized that the application of electronic devices and platforms can enhance multidisciplinary communication, collaboration, operational support, and patient involvement, potentially improving healthcare service efficiency and reducing costs (1–7). These technologies and the services they offer are commonly referred to as digital health innovations (DHIs), whereas healthcare digitalization denotes the broader transition toward embedding digital tools into daily work practices (8). Examples include, among others, electronic patient records, communication platforms, decision support software, artificial intelligence, electronic prescription services, and e-health services such as telemedicine and remote consultations.

The process of health digitalization is difficult and failure-prone (4, 5, 9, 10) and, while theories, models, and frameworks (TMFs) exist to describe and direct this process, there is little guidance to their appropriate selection and application. In this review, we aim to unpack these TMFs and the contexts and foundations of their development to create a holistic picture of how the TMFs differ and relate to one another. It should be noted that the concepts of “theory”, “model”, and “framework” are used interchangeably in both literature and practice, despite the fact that the real meanings of these words are different (11–13). Disentangling the technical differences between these terms is beyond the purpose of this review and therefore we will group them under the abbreviation TMF.

There is a vast amount of published TMFs in the domain of implementation research (14). For example, in their systematic review, Heinsch et al. (2021) identified 36 TMFs commonly used in implementation science, which had been applied across 119 eHealth projects. Among these, Davis's Technology Acceptance Model (TAM) and its successor, the Unified Theory of Acceptance and Use of Technology (UTAUT), along with the Normalization Processing Theory (NPT) and structuration theory, were the most frequently used. However, these TMFs have been widely criticized in the literature by opposers arguing that health digitalization encompasses more than the implementation of isolated DHIs. Although the TAM and UTAUT are orientated toward technology adoption and acceptance, they were not originally developed for the healthcare setting (15) and have consequently been criticized for overlooking healthcare-specific cultural, regulatory, and organizational factors (16). The NPT, while developed within the healthcare domain for complex interventions, does not specifically address technology and data concerns (17) such as technological anxiety, privacy and security matters, and technical complexities requiring additional expertise (4). Therefore, there is a need to focus on the appropriateness and use of TMFs specifically developed to bridge the domains of healthcare implementation sciences and technology adoption.

Existing TMFs require revision and adaptation to address the changing and complex culture of healthcare systems, digital technologies, and data management. These newly developed TMFs are mainly based on *a priori* theorization and empirical research. There are diverse reasons and justifications for including certain constructs and factors in contemporary health digitalization TMFs and excluding others. These reasons are built upon underlying assumptions about what is believed to be true in a certain situation or context. Justification is the action of creating logical connections between assumptions and reasons. Underlying assumptions reflect the context, experiences, and priorities of different actors within the digital ecosystem. In addition, TMFs differ in their focus regarding the stage of the digitalization process they address and the purpose of the DHIs they aim to support. The result is a heterogeneous constellation of TMFs that reflects the multilevel and multidisciplinary nature of health digitalization yet also contributes to fragmentation and confusion regarding the value and appropriate use of each TMF.

In this systematic review, we aim to provide an overview of the different health digitalization TMFs developed from empirical research. We will go beyond thematically analyzing and synthesizing extrinsic TMF constructs to create a unique fingerprint of each TMF. This unique fingerprint relates to the role and purpose of each TMF, their context, method of development, and intrinsic and extrinsic value. In addition, we will thematically analyze the assumptions about healthcare systems that are intrinsically embedded into the different TMFs. The elements of this fingerprint can help guide different actors to selecting appropriate TMFs and maximize the value of integrating TMFs into their health digitalization strategy.

## 2 Methodology

### 2.1 Registration details, declarations, and funding

The study protocol was preregistered in PROSPERO under registration PROSPERO 2025 CRD420251027510 and can be found at https://www.crd.york.ac.uk/PROSPERO/view/CRD420251027510. The PRISMA 2020 guidelines were used in structuring this systematic review report.

### 2.2 Search strategy

A systematic search was performed in the databases of PubMed, Embase.com, and Clarivate Analytics/Web of Science Core Collection. An information specialist (GB) searched the databases from inception to 25 February 2025. The search included keywords and free text terms for (synonyms of) ‘theoretical model’ combined with (synonyms of) ‘Digital Technology’ combined with (synonyms of) ‘Stakeholder Participation’. No limitations on date or language were applied in the search. We also identified key words from relevant articles and consulted the MeSH term directory from PubMed for suitable alternative terms and synonyms. A full overview of the search terms per database can be found in the Supplementary Information (see Supplement A).

### 2.3 Inclusion and exclusion criteria

Studies were eligible for inclusion if they were published after 2000, written in English, and presented a new or significantly adapted (to the healthcare context) TMF tested in an empirical healthcare setting. Studies needed to include both internal and external actors such as technology innovators, developers, or vendors involved in the innovation life cycle of a disruptive DHI. Studies applying only a known TMF without amendments to the DHI context or TMFs not supported by empirical evidence were excluded. TMFs presenting a digital innovation in the prototype phase were also not eligible. We excluded TMFs that did not address digital health innovation, business/financial models, and health service models, as these could not explain how healthcare systems influence health digitalization. Studies not considered middle-range theories according to the definitions of Peterson & Bredow (18) were also excluded for the same reason.

### 2.4 Screening and selection

The first round of pilot screening was done wherein two researchers (CM and HM) independently screened the same 300 articles. Thereafter, a training session was conducted to refine inclusion and exclusion criteria and align screening methods. The same researchers independently screened all titles, abstracts, and full texts from the whole dataset. Disagreements regarding including or excluding particular studies were discussed with the research team (CM, HM, MdB, and MM) during consensus meetings.

### 2.5 Data extraction and coding

Manuscripts were first carefully read several times to familiarize ourselves with the data. CM wrote short narrative summaries of each included study that was then presented and discussed for accuracy and appraisal by the research team.

The aim of this systematic review, together with the appraisal criteria developed by Peterson and Bredow (2020), guided the formation of topic codes. Topic codes comprised the following components: purpose of the TMF; underlying theories or foundational models; method of TMF development; context in which the TMF was developed; type and method of data collection; research population; TMF outcomes; included constructs; construct definitions; construct relations; method of analysis; and takeout messages based on the TMF.

Coding was conducted in two stages. In the first stage, one researcher (CM) coded sections from the text based on the above data categories using MAXQDA. During the second stage of coding, text pertaining to constructs, the definitions, justifications, and background information of the constructs, as well as the purpose and rationale of the new TMF, were isolated. We used latent coding to label the abstract higher conceptual interpretations of how the authors perceived the healthcare landscape and the aspects they considered important during TMF development. By using latent coding, we were able to abstract embedded assumptions that needed to be true in order for the proposed TMF constructs (and their justifications) to be true. For example, the coded segment “*In highly competitive markets, IT innovation adoption is necessary to maintain and achieve competitive advantage*” was initially coded as “healthcare providers compete with one another”. Through interpretive and reflexive analysis, we supposed that this statement can hold value within the healthcare sector only if healthcare functions as a commodity wherein consumers are free to choose their provider based on market logics. Hence, the latent code “healthcare functions as a commodity” was assigned to this section.

A matrix containing the coded segments of texts and inferred assumptions was discussed in depth during consensus meetings (CM, MdB, HM, and MM) in order to verify the links between coded segments and the identified inferred assumptions. Where disagreements existed, the research team used existing literature on the nature of healthcare systems to guide their decision.

CM extracted the rest of the topic codes from the included studies into a comparative matrix. After data extraction, we sought additional information regarding the type of healthcare system and its payment structure in the country of TMF development. The rest of the topic codes, and additional information, were used for contextual understanding and critical appraisal of the data.

### 2.6 Data analysis and synthesis

All included studies were retained for synthesis and analysis, as unsuitable studies were already excluded during the screening process.

In order to better understand the differences and similarities in perspectives of the included TMFs, we investigated the scholarly origins and background of each TMF. The origins and foundation theories for each group of TMF were mapped using alluvial charts.

We grouped individual constructs and factors from the TMF according to their position in the digital ecosystem and proximity to the DHI. In this paper, we will refer to abstract and complex ideas or phenomena as “constructs”, while the term “factor” will be used for concrete measurable variables influencing constructs. Groups were drawn from key publications in organizational and implementation sciences (19, 20). These system level groups include the individual level (micro), inner setting (meso), and outer (macro) setting. Because of the multidisciplinary and multiorganizational nature of the ecosystem, we differentiated the individual level into three subgroups, namely the end-user, the innovator/developer of the DHI, and the characteristics of the technology itself. The meso or inner-level pertains to organizational processes and systems. We needed to differentiate between healthcare organizations and the organizations of technology innovators/developers. The macro level or outer setting refers to national policies and wider context constructs. Macro-level constructs include regulatory constructs and societal constructs. Regulatory constructs are constructs related to healthcare system regulations, policies, and laws, while societal constructs include social developments and trends.

We identified inferred underlying assumptions from the data during the inductive phases and coded them accordingly. During the deductive phases, we thematically organized coded assumptions into categories by comparing them on conceptual similarities and their functional domain within the healthcare system. Our approach differed from standard thematic synthesis by focusing on implicit assumptions embedded in the TMFs rather than identifying explicit themes across studies and constructs. We thematically analyzed and synthesized coded sections related to lessons learnt and recommendations by means of constant comparison with other TMFs and other DHI implementation guidelines.

### 2.7 Critical appraisal of TMFs

CM used the criteria from Peterson and Bredow (2020) to analyze the intrinsic and extrinsic values of each TMF. We examined the intrinsic value of the theory by evaluating its plausibility, logical reasoning, truth of built-in assumptions, the quality and type of data upon which the theory is built, and its understandability. We specifically looked for properties such as clarity, consistency of terms, and how easy the TMF is to follow and comprehend in the intrinsic analysis. For the extrinsic value analysis, we focused on the appropriateness and usefulness of the TMF. During the external value analysis, we considered the TMF's complexity, uniqueness, alignment of underlying assumptions to the current context, pragmatism, scope, and significance. We considered good extrinsic value almost like one would consider virtues. These are properties wherein the saying “more is not always better” rings true. For instance, looking at the scope of a theory, the literature argues that a too narrow scope disregards too much of reality for the theory to be practical, while a too broad scope becomes an unmanageable amount of information to handle efficiently and clouds the purpose of the theory (11, 13, 18). The rest of the research team reviewed and discussed the analysis.

## 3 Results

### 3.1 Study selection process

**Figure 1:**
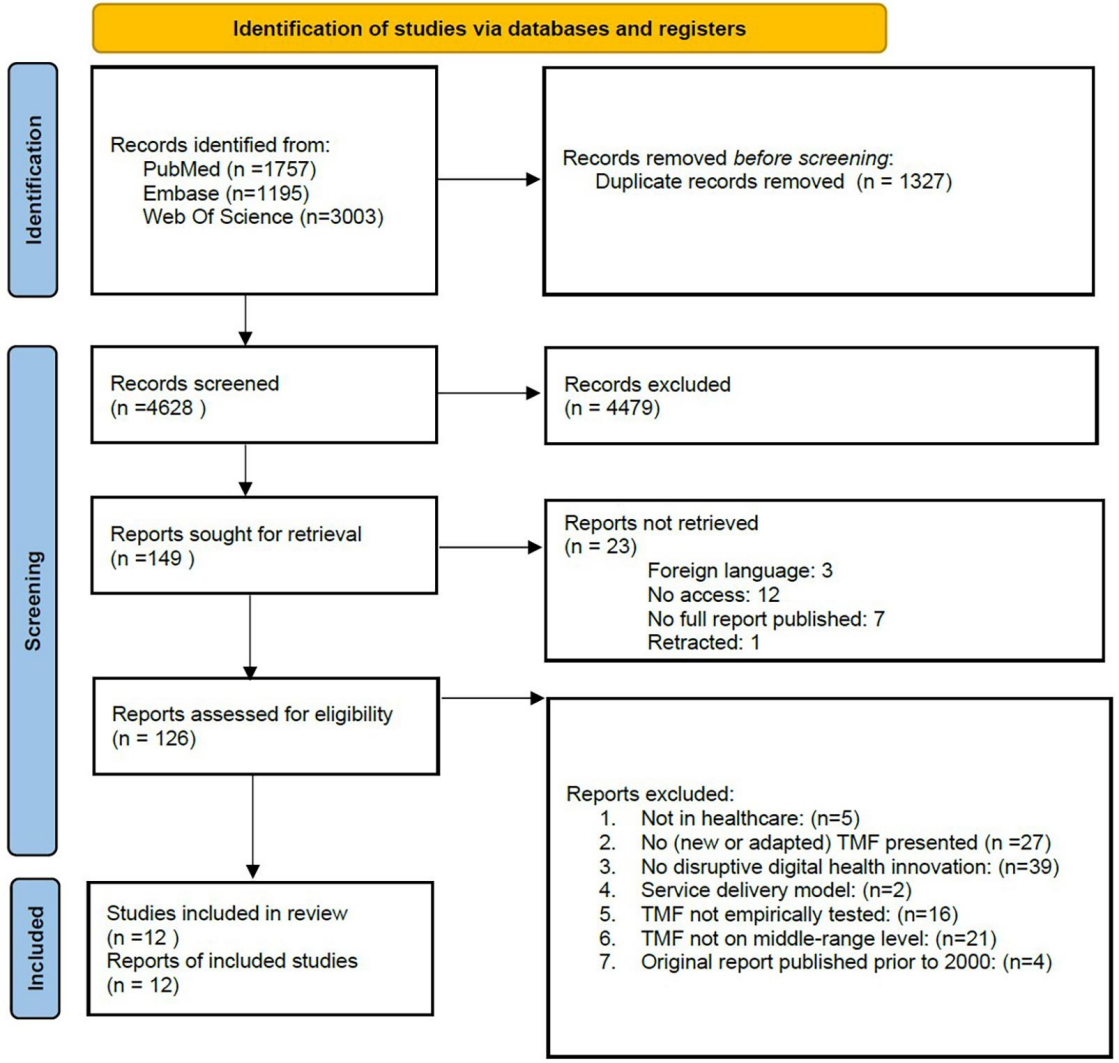
PRISMA flow chart.

Figure 1 shows our PRISMA flow chart. Our search resulted in 4628 identified scientific records after duplicates were removed. A total of 126 full text reports were screened, of which 12 studies were eventually included in the final report. We present the reasons for excluding the remaining 114 reports in Figure 1 (PRISMA diagram). The included studies were published between 2011 and 2024, with 8 studies published after 2020.

### 3.2 Goal, purpose, and outcomes of the TMFs

Through thematic analysis, we identified three groups of TMF based on their intended outcomes or dependable variables. Group 1 (*n* = 5) consisted of a TMF aiming to predict or explain the factors leading to behavioral change among end-users measured either as the intention to use the DHI or actual use of the DHI (Beglaryan, 2017; Yousef, 2021; Byrd, 2021; Zhang, 2024 and Jin, 2024). Group 2 (*n* = 4) focused on the factors influencing organizational adoption and support of the DHI (Harvey, 2012; Nilashi, 2016; Greenhalgh, 2019 and Deng, 2021). The primary objective of Group 3 (*n* = 3) was to describe and explain the influencing factors or methodologies for the design and development of a DHI (Boussadi, 2011; Nikayin, 2013 and Pietronudo, 2022).

### 3.3 Origins of the TMFs

The studies in groups 1 and 2 stem from a positivist paradigm measuring the existence of constructs and their effect on outcomes which, in this case, is intention to adopt a new behaviour (sustained use of the DHI) and organizational adoption of the DHI respectively. Studies focusing on technology design and development (group 3) have followed a more pragmatic view focusing on strategies and practical steps to support value-based DHI design and development. These TMFs included emphasis on improving user-centeredness, strengthening infrastructure, satisfying end-user needs, and building a supportive environment.

**Figure 2:**
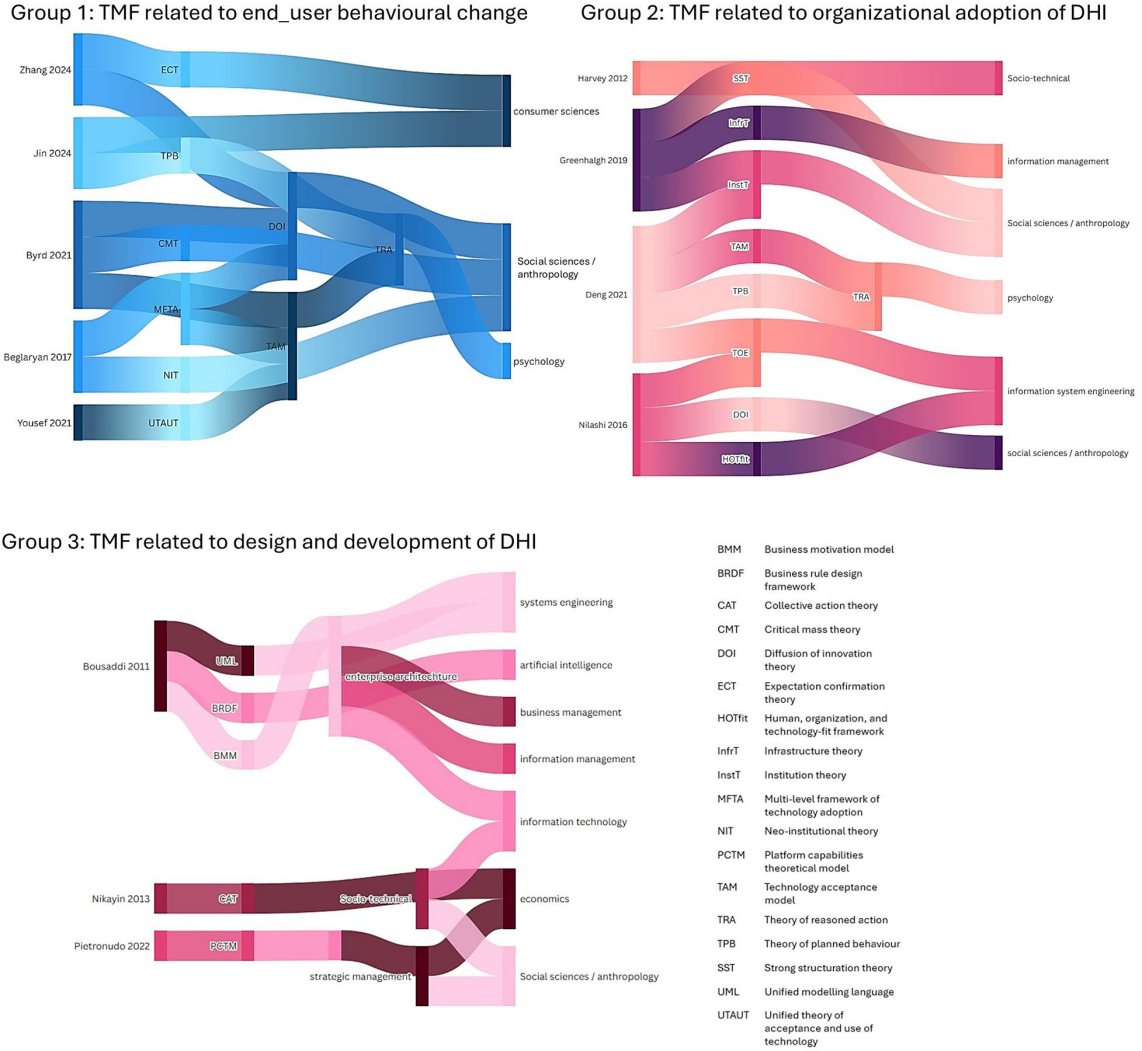
Scholarly domain origins and foundational theories of TMFs in health digitalization.

The included TMFs were developed by combining constructs from a range of scientific domains outside healthcare, reflecting the heterogeneity of included TMFs and the complexity of the healthcare digitalization phenomena. The majority (*n* = 11) of them were developed by formulating a hypothesized TMF based on the literature and then testing it in an empirical setting. Only Harvey, 2012 used an inductive approach to build their TMF while supporting their TMFs with assumptions and prior knowledge from the sociotechnical domain. Figure 2 gives an overview of how different domains and foundational theories were combined in the development of the TMFs.

Through careful analysis of the texts, we abstracted the underlying perspective, rationale, and objective that informed each study as presented in Table 1. Table 1 also shows the foundational theories of the TMF and research methodologies used to create and test associations within.

**Table 1:** Overview of the characteristics of TMFs related to health digitalization.

STUDY CODE	Beglarian, 2017	Yousef, 2011	Byrd, 2021
TMF OBJECTIVE AND FOCAL CONSTRUCTS	Determined the influence of computer anxiety, organizational support, administrative monitoring, professional relationships, personal innovativeness and resistance to change on behavioral intention.	Determined the (lack of) moderation effect of age, professional role and years of professional experience on the relations of the UTAUT theory.	Determined the influence of perceived critical mass on behavioral intention in different stages of innovation diffusion.
TYPE OF THEORY	Predictive theory	Predictive theory	Predictive theory
ASSIGNED GROUP	Behavioral change TMF	Behavioral change TMF	Behavioral change TMF
SETTING OF DATA COLLECTION	Hospitals (multi, setting) in Armenia	Hospitals and primary healthcare clinics (multi-setting) in Saudi-Arabia	Inpatient and clinic departments in two hospitals (multi-site) in the USA
TYPE AND PURPOSE OF DHI	National electronic health record (EHR)	Secure messaging feature linked to a personal health record (PHR) platform	HIPPA-compliant Voceral Collaboration Suite (Smartphone application) that enables safe messaging and voice calls on private smart phones
RESEARCH METHOD	**Data collection:** Paper-based survey conducted with hospital physicians (*n* = 233) **Data analysis:** ESEM analysis	**Data collection:** Online survey sent to multi-disciplinary health care providers (*n* = 224) **Data analysis:** SEM analysis	**Data collection:** Survey distributed to physicians and nurses (*n* = 726) **Data analysis:** SEM analysis
FAUNDATION THEORIES USED	TAM	UTAUT	TAM
			DOI
			Critical Mass Theory
UNDERLYING PERSPECTIVE AND RATIONALE	Social influence (patient and professional relations) and organizational barriers guide end-user perceptions, especially perceived collective usefulness Physician autonomy is a driver of resistance to change Projected collective usefulness forms a bridge between perceived usefulness and behavioral intention	Individual perceptions are socially and organizationally shaped Different specializations and individuals with different levels of seniority have different roles and thus different needs and priorities of DHI Age may potentially be a catalyst for inter-individual differences	The value of DHI's is dependent on their collective use Perceived critical mass plays a major role in technology acceptance and utilization through its influence on perceived usefulness, perceived ease of use and behavioral intent

**Table 1:** Continued

STUDY CODE	Zhang, 2024	Jin, 2024	Harvey, 2012
TMF OBJECTIVE AND FOCAL CONSTRUCTS	Determined the effect of network externalities on behavioral intention through their influence on satisfaction	Determined the effect of TPB constructs and perceived service quality on behavioral intention, and electronic-word-of-mouth through their influence on satisfaction	Identified and described how specific organizational factors (workways) shape successful adoption of DHI
TYPE OF THEORY	Predictive theory	Predictive theory	Descriptive theory
ASSIGNED GROUP	Behavioral change TMF	Behavioral change TMF	Organizational adoption
SETTING OF DATA COLLECTION	Online healthcare consumer community in China	Online healthcare consumer community in China	Multi-site community pharmacies in the UK.
TYPE AND PURPOSE OF DHI	Online health platforms for the management of chronic diseases. Features include healthcare information, social support, sharing health and healthcare experiences, and addressing dissatisfaction with the information received during in-person consultations	Platforms for telemedicine, including features of e-prescriptions, remote monitoring, access to electronic health records, AI-based symptom check, health education, appointment scheduling, mental health support, pharmacy services, health insurance services or limited urgent care	Electronic prescription service for processing and dispensing medicine
RESEARCH METHOD	**Data collection:** Questionnaires distributed to patient users of the platform (*n* = 518) **Data analysis:** SEM analysis	**Data collection:** Electronic questionnaire distributed to patients and healthcare providers (*n* = 593) **Data analysis:** SEM analysis	**Data collection:** Multi-site ethnography and interviews with pharmacists (*n* = 15) **Data analysis:** Inductive thematic analysis
FAUNDATION THEORIES USED	DOI Expectation Confirmation Theory	TPB	Theory based on inductive analysis of the data
UNDERLYING PERSPECTIVE AND RATIONALE	Healthcare provider's perceptions are shaped by patient perceptions, needs and satisfaction, underlining patient centredness in healthcare DHI implementation should be beneficial to the whole DHI eco-system, including health providers, patients and other health organizations. The benefits of DHI implementation to ecosystem can be framed in terms of network externalities	Healthcare providers’ perceptions are shaped by patient perceptions, needs, and satisfaction, underlining patient centeredness in healthcare DHI need to be convenient for consumers in order to be sustained and supported by healthcare providers	Managerial processes, work organization, internal structures, social and technological interactions also play a crucial role in DHI adoption and integration There is a reciprocal relation between work processes, technology and social interactions Internal structures, organizational and social processes needed to be adapted to accommodate technology

**Table 1:** Continued

STUDY CODE	Nilashi, 2016	Greenhalgh, 2019	Deng, 2021
TMF OBJECTIVE AND FOCAL CONSTRUCTS	Determined the relative importance of TOE, HOT-fit and DOI constructs in hospital management's decision to adopt or support an innovation	Described how infrastructure characteristics shape adaptation of DHI during the process of technology adoption	Developed a framework, and associated evaluation tool, to study contextual factors affecting innovation diffusion and adoption across an organization
TYPE OF THEORY	Predictive theory	Descriptive theory	Descriptive theory
ASSIGNED GROUP	Organizational adoption	Organizational adoption	Organizational adoption
SETTING OF DATA COLLECTION	Public hospital management departments (multi-site) in Malaysia	Outpatient departments (multi-site) in the UK	Urban and specialist regional medical consortiums of Primary, Secondary, and Tertiary (multi-site) medical institutions in China
TYPE AND PURPOSE OF DHI	Hospital information system (HIS) with clinical, administrative and financial modules	Video-call service linked to mobile phone application for tele-consultations	Point-of-care test for cancer screening (Des-Gamma Prothrombin test)
RESEARCH METHOD	**Data collection:** Questionnaire distributed to senior hospital management (*n* = 20) **Data analysis:** Pairwise comparison and fuzzy analytic network process (ANP).	**Data collection:** Case study description comprised of 4 sub-case studies. Ethnographic field observations (*n* = 470 h), interviews (*n* = 111), document review (*n* = 60) and other artifacts **Data analysis:** Deductive thematic analysis	**Data collection:** Questionnaire distributed to healthcare providers (*n* = 246) **Data analysis:** SEM analysis
FAUNDATION THEORIES USED	DOI	Strong Structuration Theory Infrastructure Theory	TAM
	TOE		TPB
	Institution theory		TOE
	HOT-fit		Institution theory
UNDERLYING PERSPECTIVE AND RATIONALE	Hospitals are businesses operating in an institutional environment and DHI is a managerial process Technological and environmental factors are the most important drivers followed by organizational and human factors in organizational DHI adoption decisions	The healthcare sectors are complex, heavily regulated and ‘fast paced’ Digital infrastructure and mobilization of organizational support are crucial components of DHI adoption and implementation Digital infrastructures need to be dynamic, accommodating both the individual and managerial processes of healthcare organizations	Diffusion is inherently a social process which is shaped by individual, organizational and environmental influences including personal beliefs, technical drivers, organizational willingness to share and receive information and industry competition pressures. These constructs can be measured in order to measure organizational readiness for DHI adoption.

**Table 1:** Continued

STUDY CODE	Boussadi, 2011	Nikayin, 2013	Pietronudo, 2022
TMF OBJECTIVE AND FOCAL CONSTRUCTS	Validated a design method using Unified Modeling Language (UML) when shaping the semantics and content of DHI	Determined the effect of collective action theory constructs on co-creation efforts of a digital health platform	Described the influence of platform owner capabilities on innovation characteristics and diffusion
TYPE OF THEORY	Prescriptive theory	Predictive theory	Explanatory theory
ASSIGNED GROUP	Design and development	Design and development	Design and development
SETTING OF DATA COLLECTION	Single-site hospital-based pharmacy in France.	Health care platform eco-system in Finland	Health care platform eco-system in Italy
TYPE AND GOAL OF DHI	Clinical decision support plug-in for pharmaceutical validation and safety alerts	A platform that integrates data from several independent living assistant devices enabling remote monitoring	An AI-embedded direct access platform that allows (healthcare) data sharing while maintaining privacy and security of the data
RESEARCH METHOD	**Data collection:** Case study description from authors’ own experience **Data analysis:** Deductive thematic analysis	**Data collection:** Case study description. Stakeholder interviews (*n* = 10), project documents and reports, scientific publications **Data analysis:** Deductive thematic analysis	**Data collection:** Case study description comprised of semi-structured interviews (*n* = 2) company documents and online available information **Data analysis:** Deductive thematic analysis
FAUNDATION THEORIES USED	Semantics of Business Vocabulary and business Rules (SBVR) model	Collective Action Theory	Helfat and Raubitschek platform capabilities theoretical model (2018)
	Business Rule Design Framework (BRDF)		
UNDERLYING PERSPECTIVE AND RATIONALE	Successful implementation depends on how well the innovation integrates with the current work practices of an organization. A one-size-fits-all approach is ineffective Business process management techniques can be used to create adaptable, tailored DHI solutions considering individual-, organizational-, and industry-level needs	Successful digitalization in healthcare contexts depend on collaboration between multiple actors Factors such as technology platform openness, selective incentives and heterogeneity of interests and resources are instrumental to eco-system collaboration. Leadership is crucial to collaboration by protecting and promoting individual actor interests and selective incentives	Platforms can support the health care sector by facilitating learning, networking and increasing organizational capacity. Strict regulation of the healthcare domain leads to information and innovation fragmentation preventing large-scale collaboration. Platform owners play an instrumental role in facilitating interorganizational collaboration by sensing industry technology needs, innovating and integrating solutions

The academic fields of psychology, social sciences, anthropology, and consumer sciences form the foundation of TMFs focusing on behavioral change and organizational adoption (groups 1 and 2). Roger's Diffusion of Innovation theory (DOI) and Davis's Technology Acceptance Model (TAM) were most frequently incorporated into TMF groups 1 and 2. The DOI theory and TAM were complemented by constructs arising from other theories such as the critical mass theory, institutional and neoinstitutional theory, theory of planned behavior, technology–organizational–environment (TOE) framework, and human–organization–technology fit (HOT–fit) framework.

All of the studies in group 1 stated that an individual's perception of DHIs play a determinative role in DHI implementation, acceptance, and use. The authors investigated individual, social, and organizational driving forces of individual perceptions. Individual driving forces included age, level of seniority, and professional role. Social influences investigated pertained to professional relations, patient perceptions, perceived critical mass, and projected collective usefulness, while organizational factors focused on physician autonomy, network externalities, and organizational change.

The four studies in group 2, focusing on organizational adoption of DHIs, combined the origins of group 1 TMF [psychology and social sciences, specifically the theory of reasoned action (TRA)], with origins from TMF group 3 (information management, sociotechnical sciences, and systems engineering). These studies particularly focused on how organizational resources, organizational culture, and environmental (regulatory and market) factors shape managerial support and top-management decisions to adopt DHIs. Overall, these studies stress the importance of technology compatibility and adjustability to organizational contexts.

The included studies in TMF group 3, focused on technological design and development, drew from a more heterogeneous foundation that included constructs embedded in systems engineering, studies in artificial intelligence, information management and economics, and social sciences and anthropology. These studies collectively emphasize the importance of providing support to multiactor collaborations and designing technological solutions that integrate with end-user needs.

### 3.4 Placing TMFs in context

As mentioned, it is often difficult to select a TMF to support DHI projects. A number of aspects determine the appropriateness of a TMF in real-life projects, such as how well the TMF aligns with the context, the actors involved, the objectives for using a TMF, as well as the intrinsic and extrinsic value of a TMF. In the following section, we will map the contexts of the included TMFs in order to support the reader in selecting and appropriately using these TMFs. Table 1 further includes the main characteristics and context of the included studies, their purpose, inherent perspective, context, and foundation.

The type of national healthcare model in which the TMF was developed and tested has vast implications on health digitalization as it concerns the influences of authority, autonomy of healthcare professionals, decision-making and adoption processes, regulatory frameworks, work organization, responsibilities of healthcare professionals, and innovation financing. The included TMFs came from an array of different healthcare systems, with the majority (*n* = 8) based on empirical evidence conducted in the national healthcare settings of the UK, China, Finland, Mexico, Spain, Italy, and Saudi Arabia, all of which have a national health insurance model wherein healthcare professionals are paid a monthly fixed salary by a healthcare institution and the institution decides whether or not to adopt a technology. However, differences do exist between the healthcare systems of these countries; for instance, of these, Italy and Finland have a more regional decision-making structure. The healthcare systems of Malaysia and Armenia are tax-funded for basic healthcare but rely on individual contributions for extended healthcare benefits and improvements. The US, on the other hand, has a private insurance dominant healthcare system significantly funded by individuals (32). There are also vast differences in how much the government (tax or other national income) contributes to the healthcare sector, with the UK having the largest percentage of government contributions and Armenia the lowest (33).

### 3.5 Assessment and critical appraisal of TMFs

According to Peterson and Bredow (2020), TMFs differ in terms of their intrinsic and extrinsic value. Intrinsic value relates to how TMFs are developed by evaluating the authenticity of the data used, clarity, consistency, logic, and level of theory development. Extrinsic value examines the usability of a TMF and considers the complexity, uniqueness, truth of underlying assumptions, pragmatism, scope, significance, and utility.

Not all TMFs are equally valuable in every context; selecting the most appropriate theory for a given application requires careful judgment. Supplement B depicts the major differences between the TMFs based on the extensive criteria by Peterson and Bredow, which are used to guide both internal and external criticisms of TMFs.

Studies aimed at predicting or explaining end-user behavioral change (TMF group 1) were mostly uncomplex with varying degrees of scope and abstraction. Byrd-2021 had lower levels of abstraction, while Zhang-2024 presented a much broader scope.

Studies focusing on organizational adoption (TMF group 2) were mostly of a broader scope and higher abstraction, except for Harvey, 2012. This makes them more overarching and adequate but also more difficult to incorporate into design or dissemination strategies.

In the group of studies related to technology design and development (TMF group 3), the TMFs were more complex. Although a lower level of abstraction, the work by Boussadi, 2011 entailed many steps and related artifacts, while Pietronudo, 2022 entailed complex relations between independent and dependent variables and the model by Nikayin, 2013 used complex constructs.

### 3.6 Constructs and factors show different assumptions of the health digitalization landscape

The value of using a TMF is highly dependent on its intrinsic embedded assumptions, underlying its purpose, constructs and relations, holding true within the context (18). Assumptions form the basis of perspectives and point of views, hence guiding argumentation, evidence generation, and selection of research methodologies and forming conclusions (34). They are beliefs about how the world works and shape our understanding of what is important and why. We chose to label these underlying ideas as “assumptions” because they were not explicitly defined or based on scientific literature by the authors. Rather, they were ideas that the authors took for granted and emerged through their hypothesis, conceptual frameworks, and rationales. This is in line with the theoretical definition and explanations regarding the concept of assumptions (34).

**Figure 3:**
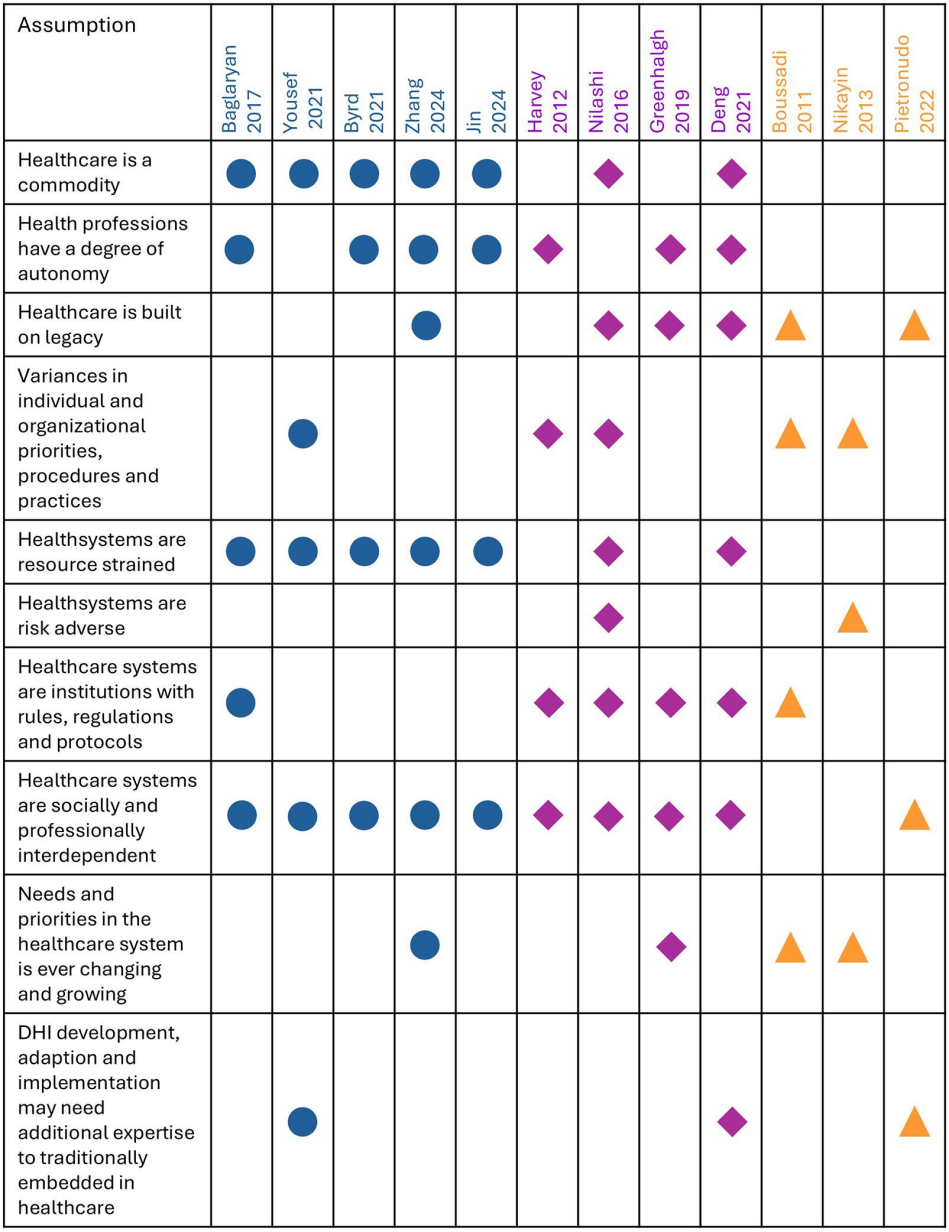
Distribution of health system assumptions across TMFs groups.

Through an in-depth dissection, latent analysis, and discussion of each TMF, their origin, constructs, justifications, and relations, we identified the embedded assumptions that need to be true in order to support the claims of the TMF. These assumptions were grouped according to their position within the digitalization ecosystem, namely, individual, organizational or outer setting. Ten themes emerged among the identified assumptions. Figure 3 compares the assumptions across the studies and TMF groups followed by a textual explanation of the main assumptions we have deduced from the TMF.

Within the individual (micro) setting, we have identified two assumptions embedded in seven and five studies, respectively. These assumptions related to the autonomy of health professionals and interprofessional variances in priorities, procedures, and practices.

*Health professionals have a fair degree of autonomy* was identified as an underlying assumption in TMF groups 1 and 2. This assumption is sustained by the evidence that their opinions and attitudes matter and influence the digitalization process. Studies on organizational adoption of DHIs and end-user behavioral change identified autonomy loss as a barrier to successful implementation. Deng, 2021, Jin, 2024, Byrd, 2021, and Zhang, 2024 illustrate that the perceptions, personal beliefs, and attitudes of the end-users (the health professionals) regarding the DHI play an important role, inherently meaning that they have some form of autonomy in their decision to integrate the DHI into their daily work. Beglaryan, 2017 and Harvey, 2012 stressed that maintaining this autonomy is important to the healthcare providers and that fearing that DHI implementation will cause loss of autonomy, or increase top-down control, negatively impacts DHI adoption and implementation. Greenhalgh added that when (digital) infrastructures are too rigid to support clinician autonomy, it breaks down and is rejected from the system, showing that maintaining and respecting autonomy is essential. The autonomy of health professionals, together with the universal scarcity of human resources for healthcare, means that organizational decisions, resources, and strategic alignments influence healthcare providers’ loyalty to the organization. Healthcare organizations thus not only serve as a commodity for patients but also emerge as a commodity for health professionals.

Five studies, spread over all three TMF groups, acknowledged *variances in individual priorities, procedures*, *and practices.* Boussadi acknowledges inter-organizational differences in operational processes and needs by including business process modelling in their proposed development procedure. Harvey explicitly mapped the differences in workways, work organization, and prioritization between three types of community pharmacies, showing how the differences are correlated to technology orientation and use. Nilashi and Harvey also considered the variances in resources (space, fiscal, human capacity, and infrastructure) in the development of their organizational adoption TMF. Yousef and Boussadi theorize that there are inter-professional differences in needs, priorities, and professional vocabulary that influence end-user perspectives and the design of DHI. While most of these TMF argue that individual and organizational variances create challenges for inter-operability and scale-up of DHI, Nikayin shows that the heterogeneity of resources between organizations can be beneficial to collective action and collaboration.

Within the organizational (meso) setting constructs and TMF we have identified three embedded assumptions across the dataset. The assumption that *priorities, procedures, and practices vary* was also seen on an organizational level. The other two assumptions related to perceptions that healthcare is built on past events and decisions and the social and professional interdependency of healthcare practices.

Six studies (mainly in TMF groups 2 and 3) incorporated the assumption that *Healthcare is built on legacy*. The legacy of healthcare is threefold. Design and development studies (Pietronudo- and Boussadi-) incorporated healthcare's extensive knowledge base in their TMF and consequently articulated that DHIs need a means to be updated in order to incorporate new knowledge. Greenhalg, 2019 and Nilashi- refer to legacy in terms of past decisions, investments, and existing infrastructures. Most of these past decisions did not include technological considerations, leading to issues of incompatibility, inadequate special resources, outdated technological infrastructure, and low bandwidth that will require large investments to change. Traditions or professional norms shape how end-users (both healthcare providers and patients) experience DHIs. Deng showed the predictive value of subjective norms and Zhang further argued that age and years of experience may influence end-users’ perspectives on DHI experiences through shaping professional norms and image. Both these studies reflect that technological orientation is, despite advancements in digital health, not yet considered the norm within healthcare.

Multiple TMFs (*n* = 10) share a common view that DHI implementation is somehow linked to social processes, leading to the assumption that *Healthcare systems are socially and professionally interdependent*. In the TMFs of Deng, Nilashi, Jin, and Yousef, in addition to emphasis on individual perspectives (personal beliefs, attitudes, perceived usefulness, and ease of use), they also state that these perspectives are shaped by social interactions and perceived norms. Harvey, Beglaryan, and Pietronudo argue that social and personal interactions form an integral part of healthcare work and that health providers value these social network connections in relation to their job performance. Subsequently, the authors have added the constructs “social organization of work” and “professional relations” to their respective TMFs. Byrd, Greenhalgh, Pietronudo, and Beglaryan have incorporated constructs related to a DHI's value increasing when used collectively to support data capture and sharing, communication, collaboration, and networking. The phenomenon of social influence is also apparent on an organizational level, where organizational decisions are influenced by the actions of similar or geographically close healthcare organizations, as shown by Nilashi's “mimetic pressure” and Deng's “industry competition pressure”.

On a macro or outer-setting level, we have identified assumptions showcasing healthcare as a complex institutional setting with evolving needs and priorities, following market logics and focusing on resource and cost efficiency. Additionally, the assumption that healthcare systems have a need for external expertise during digitalization processes also emerged.

All studies within TMF group 2, along with two other studies, identified that *healthcare systems are complex institutions with rules, regulations, and protocols.* This common characteristic of the healthcare system is iterated in the works of Nilashi, Deng, Greenhalgh, and Jin, who showed the layered decision-making processes and that DHI decisions are not made at a ground-staff level. Through the inclusion of “coercive pressures”, Nilashi further acknowledges that organizational decisions are dependent on regulations and overarching political discourses. Harvey, Greenhalgh, Boussadi, and Nilashi all illustrated that operations are embedded in protocols, standards, procedures, and routines and that DHIs need to honor these in order to gain support from decision makers.

Some studies (*n* = 4) incorporated the assumption that *needs and priorities in healthcare systems are ever changing and growing.* Pietronudo, noted the importance of platform owner's “sensing capabilities” in identifying new trends, needs, and opportunities for value creation. Greenhalgh, Nikayin, and Zhang advocate that DHIs should be able to accommodate these changes either by using open platforms allowing third party providers to offer new services or by being “patchworked and patch dependent”. Boussadi also recognized that healthcare knowledge and best practices continuously evolve, requiring DHIs to be agile and adaptable to incorporate new research and standards.

Seven studies (*n* = 7) based their argument on the idea that healthcare organizations follow a market logic approach and *healthcare is seen as a commodity*. In this approach, patients are seen as clients or customers whose opinions matter, the quality and efficiency of service delivery matters, and healthcare organizations compete with one another for business. This assumption has been included in various TMF in different ways. Beglaryan, 2017 used the construct of “Patient influence”, while Zhang 2024 incorporated “Satisfaction” due to direct network externalities to show that patients are considered customers whose opinions matter. Beglaryan, 2017 and Byrd, 2021 use the term “Perceived usefulness” from the TAM, while Yousef, 2022, Zhang, 2024, and Jin, 2024 use the terms “Performance expectancy”, “Direct network externalities”, and “Perceived service quality” respectively to illustrate the need to improve service quality and efficiency for its customers. The existence of competition between healthcare organizations becomes apparent in the constructs “Relative advantage”, “Industry competition pressure”, “Intensity of competition”, and “Mimetic pressures”.

Part of incorporating market logic assumptions into TMFs is also the strong focus on cost and resource efficiency giving way to the assumption that *healthcare systems are resource-strained.* Beglaryan, Pietronudo, Yousef, and Deng concluded that organizations need adaptive capacity in order to adopt and implement an DHI. Nilashi, Beglaryan, Byrd, Jin, Zhang, and Yousef all recognize that adoption and implementation of DHI entails costs (financial, time, cognitive capacity, and disruption). They argue that a lack of adaption capacity and high costs cause resistance to change, illustrating the existing strain on healthcare system resources.

Three studies strongly underlined the considerations of costs of DHI implementation, data security (Nilashi), industry rules, and regulation. These perspectives and the strong need to justify risks give rise to the assumption that *healthcare systems are inherently risk adverse*. The emphasis on these perspectives implies that there are risks associated with DHI adoption and implementation. Deng identified that organizations may only act when the benefits outweigh the risks by including “price rationality”. Nikayin studied facilitating factors towards cocreation of a DHI and found that selective incentives and leadership are imperative in overcoming risk aversion and facilitating health digitalization. The lack of resources may be one of the causes for risk aversion, while control is a response to it. The resource shortage in health systems has further implications regarding the availability of support staff and other supporting structures such as training and education.

Lastly, the theme of *DHI development, adaption, and implementation may need additional expertise not traditionally embedded in healthcare* was also a reoccurring theme across all three TMF groups (*n* = 3). Pietronodo highlighted that platform intermediaries possess valuable knowledge and skills shown to be beneficial in health digitalization processes. Nikayin supported this idea by their findings that collective action is key to the process, and including organizations with heterogenous resources (including skills) strengthens this collective action. Nilashi, too, acknowledge that DHI implementation requires unique skills and that the existence of these skills within the organization, or as support from DHI vendors, is a major consideration prior to DHI adoption. The need for advanced technical skills contributes to the perceived costs of DHI adoption and implementation.

### 3.7 Distribution of TMFs across innovation types and actors

From the literature on DHI lifecycles as well as the included TMFs it is clear that there are a number of actors involved in health digitalization. Actors are individuals or organizations who embody a certain function within the DHI ecosystem to pursue a common goal (35). The actors involved in healthcare digitalization operate both internally and externally to healthcare organizations and include patients, healthcare practitioners, regulatory agents, support staff, administrative staff, investors and health insurance agents. Additional actors in the form of internal and external technology specialists, innovators, and vendors become relevant during the digitalization process for their expertise in digital technology, which is not inherent to healthcare. The number and diversity of actors are further increased when a DHI is implemented across healthcare provider organization borders, for instance in the case of implementing a national EHR. All the agents and actors, combined with the interactions between them, has been coined the digital healthcare ecosystem or health platform ecosystem (9–11). This ecosystem is complex and interdependent in its nature, requiring effective relations and collaboration between its actors.

As actor diversity and numbers increase, TMF focus shifts from end-user needs toward organizational strategies, competitive advantages, and institutional hierarchies. TMFs focusing on end-user behavioral change focused mostly on the perspectives of end-users, which can include healthcare professionals and/or patients. TMFs in group two extended their actor base to include innovators of the DHI (these can either be inhouse or external to the organization), technology experts (both inhouse and external), organizational managers on different levels, and national level policy makers. Non-human actors in these groups included the infrastructure and resources and regulations and policies made by national or reginal administrative bodies. Group three TMFs reiterated the same actors but with more attention to the roles of the innovators or technology experts.

**Figure 4:**
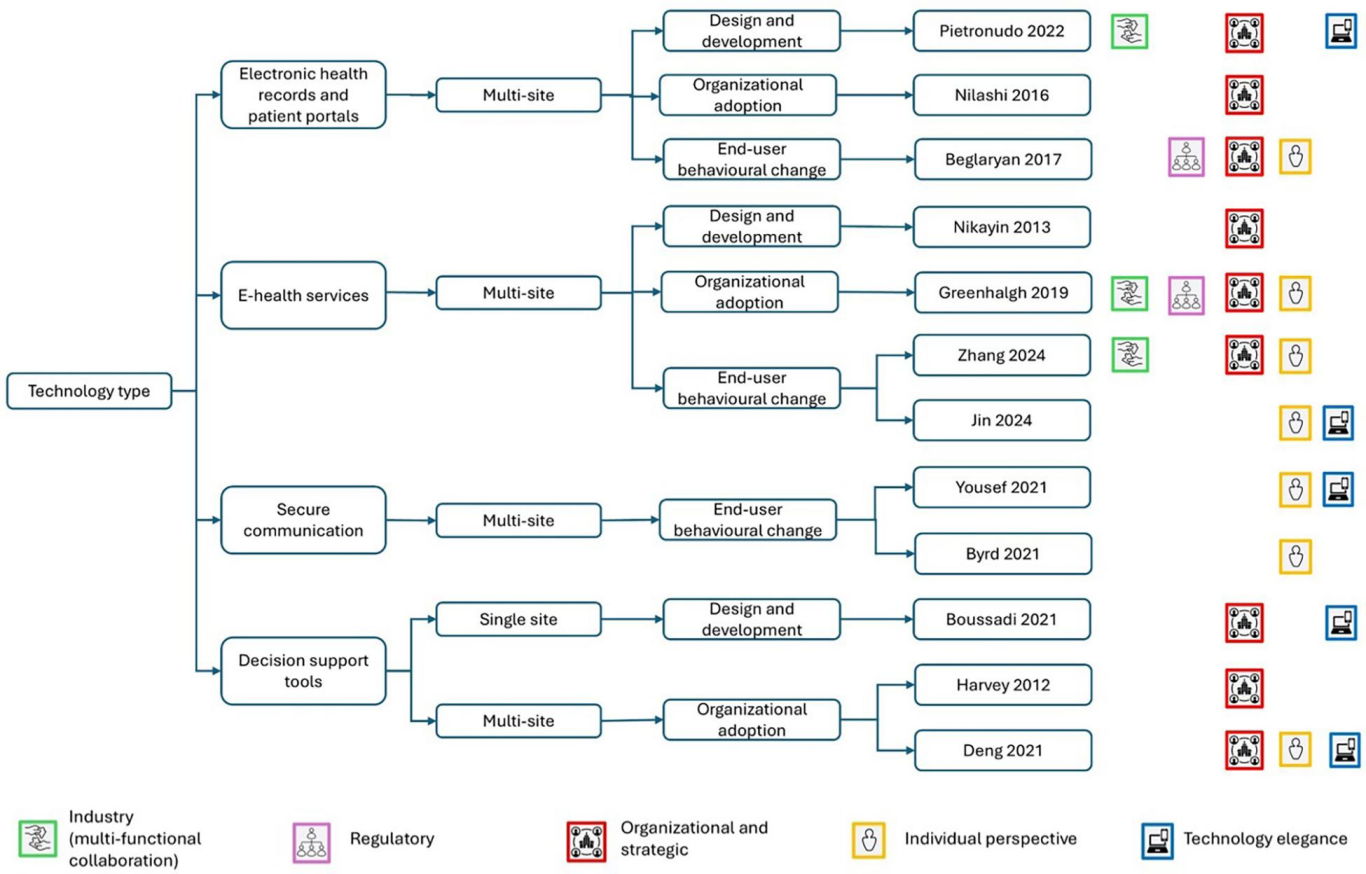
Map of TMFs by context and purpose.

We mentioned that the type of technology, the intended contexts, and the scope of implementation will impact the considerations during the different phases of the innovation lifecycle. Figure 4 is a map of the included TMFs and the context in which they were developed, which can serve as a guide as to their appropriate use. This figure also displays the system level of constructs and factors presented by the TMF.

We identified four types of DHIs that were used in the studies developing the present TMFs. The four technologies differ in their intended mechanisms of use and target audience. These differences create inherent differences in the perceived importance of success factors needed for their development, adoption, and implementation.

For instance, EHR's are used daily by health professionals but not patients and often require the health professionals to access data through fixed shared devices. The goal of such devices is to log and share information among professionals within a larger organization and are most often the result of strategic management decisions. Macro- and meso-level factors, such as stakeholder influences, individual and organizational strategic advantage, organizational resources and readiness for collective action, and security concerns are mainstream, while end-user perceptions support implementation.

Communication technologies are more for personal convenience, with each health professional having their own mobile device requiring less effort to access but its success and value depends on collective use. The TMFs focusing on communication technology reflect this by specifically highlighting social influences such as social and professional norms and perceived critical mass which, in turn, effect individual perceptions of the DHI's usefulness and convenience.

Decision support tools require embedded knowledge or intelligence, which implies additional steps in the development and maintenance of the technology. The main focus of decision support tools is to improve standards of care. Constructs related to strategic organizational advantage, organizational resources, and culture show the importance of paying attention to stakeholder interest while individual factors such as attitudes, norms, and ease of use stress that success also depends on the health care professionals’ habitual use of the tools.

Online health services are still seen as the privileged exception rather than the norm and have a greater dependence on consumer science-derived factors such as patient behavior and perceptions, user experience, and cultural fit while still being supported by the institution.

### 3.8 Actor-specific considerations, lessons learnt, and strategic insights

Ten of the included studies shared some insights as to how different actors can deal with the healthcare context and implementation barriers. These recommendations or ‘lessons learnt’ were based on the influencing factors and constructs they have identified in their TMF. In the following section we have consolidated the recommendations into a set of seven themes for recommended actions. These themes are in particular reference to key-users, technology owners or developers, and organizational managers respectively. However, it is important for all actors to take note of all themes in order to support collaboration within the ecosystem and continuity throughout digitalization projects.

#### 3.8.1 Recommendations for key end-users

Two studies (Nikayin, 2013 and Greenhalgh, 2019) highlighted the efforts of individuals (significant key-users or organizational managers) *to challenge existing regulations where necessary.*

Healthcare systems, and especially their rules, regulations, and procedures, are built on legacy and past decisions. These handed-downs are established for past specific scenarios which may not be relevant anymore. Organizational management need to adopt more flexible approaches in order to be able to realign their people, technologies, procedures, and training. More flexibility and adaptability can aid organizations to secure supporting systems and maximize value from DHIs.

#### 3.8.2 Recommendations for technology designers and developers

In reference to technology designers and owners, the data set revealed recommendations related to cultivating a teamwork approach; value creation and reducing associated risks and costs; and prioritizing DHI resilience and adaptation and working towards standardization and unification of data. These recommendations were abstracted from seven studies over the spectrum of all three TMF groups.

##### 3.8.2.1 Organizational managers and DOI innovators need to cultivate a teamwork-focused approach

Seven studies (Pietronudo, 2022; Nikayin, 2013; Deng, 2019; Greenhalgh, 2019; Zhang, 2024; Byrd, 2021; and Beglaryan, 2017) stated that digitalization processes need collaborative and coordinated efforts from multiple internal and external parties. It is important that, in order to obtain success in health digitalization, all actors need to acknowledge all assumptions and perspectives and collaborate to establish the existence of facilitating factors across the innovation lifecycle. Shared leadership may be needed to coordinate actors’ efforts and ensure continuity of the digitalization process over time. Pietronudo, 2022 specifically pointed out that platform owner's integrative capability can help cultivate teamwork and team alignment. Integrative capability is the skill to connect people and organizations on multiple levels, create a shared overarching understanding, and establish collaboration. Part of the responsibility of this common leader is to support the creation of flexible work arrangements and agreements as well as enforce selective incentives to collaboration. Leaders should also be able to create trust among stakeholders by providing stability and excellent governance.

##### 3.8.2.2 DHI developers need to focus on value creation for patients, individual end-users, and organizations

Creating value for patients, individual end-users, and organizations will aid in avoiding power struggles. The studies by Pietronudo, 2022, Nikayin, 2013, Greenhalgh, 2019, Jin, 2022, Zhang, 2024, Yousef, 2022, and Beglaryan, 2017 have suggested several strategies for involving end-users and organizations in determining value-based needs. These strategies included business process mapping, stakeholder needs and hierarchy mapping, and multiple stakeholder interviews. Additionally, DHI owners should focus on environmental scanning and anticipating needs based on current health system discourses, political decisions, and agendas to identify opportunities for value creation and actively respond to healthcare system dynamics. Organizational managers need to clearly communicate their strategic alignment and needs to all other actors to enable value creation for all stakeholders.

##### 3.8.2.3 DHI developers need to focus on reducing risks and costs associated with technology adoption and implementation

Six studies from TMF groups 1 and 3 (Nikayin, 2013; Boussadi, 2011; Jin, 2024; Zhang, 2024; Byrd, 2021, and Yousef, 2022) emphasized the need for DHI developers to reduce fiscal and implicit risks and costs associated with adoption and implementation of DHI. Implicit costs refer to time to implement and learn how to use the technology and time invested to change associated organizational policies and procedures to accommodate the technology. Interestingly, studies aiming for organizational adoption of DHI (TMF group 2) emphasized the importance of costs and price in their constructs but have not included it as recommendation in their discussion. Reducing risks can be done by focusing on the most impactful functional improvements, reducing overlap in functionalities between technologies, using existing systems where possible, and endorsing interoperability and synergy between DHIs. Usability and simplicity remains a key focus to reduce switching costs and, therefore, DHI developers need to avoid functional extravagance. Although high functionality and complex DHIs have greater potential for value creation, they also have considerably higher switching costs in the form of fiscal and implicit costs. Reducing switching costs initially allows for slow cultural changes and establishment of habits before introducing more functionalities. Thorough risk analysis and cost benefit analysis are needed in early stages to reassure organizational managers and healthcare investors.

##### 3.8.2.4 DHI innovations need to be designed and developed for resilience and adaption

The majority of studies (Boussadi, 2011; Greenhalgh, 2019; Jin, 2024; Byrd, 2021; Yousef, 2022 and Beglaryan, 2017) agreed that there are many benefits to designing adaptable DHI solutions. The lengthy multi-level decision-making processes can be reduced or avoided by designing and developing DHIs in such a way that amendments or additions to the original innovation can be made to support changing needs or grow value over time. This does imply that DHIs innovators share a long-term commitment to support and sustain innovation and organizational needs. Thorough documentation will become essential in long-term support, ensuring continuity of collaborations despite healthcare fluidity.

##### 3.8.2.5 Organizational managers and DHI innovators need to work towards standardization and unification

Heterogenous data and differences in semantics are sources of breakdown in interoperability and data sharing according to Pietronudo, 2022 and Nikayin, 2013. This was a particular pain point identified by the studies dealing with DHI design and development (TMF group 3). As mentioned, the heterogeneity stems from healthcare legacy and autonomy. It means that organizational managers need to make an effort to align their data and practices as much as possible and for DHI innovators to convert existing and new data to an “object-orientated, extensible, non-property-dependent, platform independent, international accepted standard” (29).

Some of the TMFs identified (Byrd, 2012, Zhang, 2024, Jin, 2024 and Pietronudo, 2022) in this review support the findings of Borges do Nascimento (2023) and Li & Carayon (2021) that the benefits expected from digitalized healthcare systems are highly dependable on the data end-users can access. Some authors have described this phenomenon as a chicken -and-egg situation where early adopters of technology do not yet reap the benefits of using the technology until a critical mass of users have been reached in order to use data effectively. The TMFs of Byrd, 2012 and Zhang, 2024, as well as the work of Li and Carayon (2021), argue that overcoming data compatibility or interoperability issues in the system is a key step in reaching the critical mass point.

#### 3.8.3 Recommendations for organizational managers

As discussed in the previous section, recommendations to cultivate teamwork, challenge norms and regulations, and standardize practices also have significant implications for organizational managers. There is one additional recommendation: o*rganizational managers need to prioritize both technical and organizational training.* This recommendation was supported by five studies (Boussadi, 2011; Jin, 2024; Byrd, 2021; Yousef, 2022 and Deng, 2019) across TMF groups.

Training needs to be facilitated on different levels. Boussadi, 2011 recommended that actors in implementation need to know at least one programming language in order for them to understand digitalization processes on a deeper level. Training and education on an end-user level can be used to raise awareness and reduce effort expectancy, which facilitates technology diffusion. DHI developers often play a major role in supporting organizations with their training needs, but it remains a responsibility of the organization to prioritize opportunities for training. These training opportunities should involve both technical training on the DHI as well as how to integrate the DHI into work practices and routines.

## 4 Discussion

This review aimed to create an overview of available TMF that brings together insights from research in digitalization and implementation sciences within healthcare. Our secondary objective was to guide the reader of this review in the appropriate selection and use of TMFs during health digitalization projects. We have identified and mapped out the unique fingerprint and contribution of each TMFs in terms of their purpose, context, origins, actors, and embedded assumptions. Careful consideration of this fingerprint and how well it represents the intended digitalization project is paramount to selecting and adapting TMF (18).

### 4.1 Tensions in healthcare landscapes arise from contradicting and paradoxical assumptions

Through careful deliberation and deep reflection of the assumptions and literature, we have identified some key paradoxes and contradictions in how the healthcare landscape is perceived. These contradictions and paradoxes lead to gaps between health digitalization ideology and reality, also explaining many of the previously identified barriers to DHI implementation. We will use the findings of a meta-literature review regarding barriers and facilitators to health digitalization to illustrate the impact of these paradoxes on digitalization processes within the healthcare sector. In this review, the authors identified six areas of issues: challenges with infrastructure, lack of training and education, legal and ethical concerns, time and work-related concerns, personal and psychological reasons to resistance, and poor fit or poor quality of the technology (4).

The first major tension relates to ‘Risk adversity’ and can be linked to many of these points of friction as it forms part of the legacy of healthcare and plays a major role in institutionalizing healthcare. These three assumptions (risk adversity, legacy, and institutionalism) are in contrast with assumptions such as healthcare providers have changing needs and autonomy and variances in professional and organizational processes and priorities. The first group tends to be more conservative and stability-orientated while the latter is more dynamic and flexible. Actors associated with technology design and development acknowledge the changes in healthcare needs while considering what is already part of the legacy. In contrast, TMFs focusing on organizational adoption seem to place more focus on static dimensions such as resource scarcity, hierarchy, rules and regulations, and existing structures. Our result supports the prior work of Tampio et al. (2023) in which the authors discussed the consequences of changing needs, knowledge, priorities, and people within the rigidity of healthcare institutionalism. The authors found that this contradiction causes uncertainty due to the impossibility to know the exact current or future states of the system and relations within. Uncertainty implies risks for innovators and healthcare organizations alike, something that is at friction with the identified assumption that healthcare systems are risk adverse.

It is possible that the tension between healthcare systems’ risk adversity and the changing needs of its actors lead to increased institutionalism in an attempt to create stability. For instance, to compensate for the risks, developers of innovation lifecycle models within the healthcare setting have included additional requirements such as licensing (37, 38), pre-clinical trials (38), and ensuring sustainability and governance of the innovation. Innovation implementation is sometimes split into a phase for organization adoption (that is the decision of health organizations and institutions to support the use of the innovation) and behavioral acceptance of the end-user through diffusion and dissemination. Due to the institutional and regulated nature of healthcare, even if an DHI is spread through diffusion and social means, organizational adoption is still required. The process of diffusion is sometimes actively inhibited by organizational structures, rules, and regulations, as shown by Greenhalgh's 2019 case study. Legal and ethical concerns arise from the differences between fast-paced technology development and the slow pace of health innovation testing and accreditation associated with the risk adverse nature of healthcare.

Tension also plays out in the matter of autonomy vs. institutional control. While several constructs within the end-user behavioral change models point to healthcare professionals’ autonomy and the importance of their perceptions in choosing DHIs, other constructs related to organizational adoption and technology design and development recognizes the strong hierarchical and regulated nature of healthcare systems. This tension manifests itself in the common barriers identified and gives rise to power struggles often described within the sector regarding DHI adoption and implementation. Beglaryan 2017 acknowledges the tension between the two assumptions in their construct “Administrative monitoring” and their explanation that it captures “physician's belief that using EHR will increase top-down monitoring over their practices”. Legacy in the sense of perceived professional norms, together with concerns regarding loss of autonomy, can be considered personal psychological reasons for resisting DHI implementation.

The institutional nature of health systems means that DHIs are often selected by top management according to organizational priorities and strategies. When the selection is made improperly, the result is technology that does not align with the needs and priorities of the autonomous healthcare provider. Nilashi 2019 considers finding this balance an important factor to consider in organizational adoption of DHI through the inclusion of the construct “compatibility”, and Deng 2021 refer to it as consideration to personal beliefs.

Healthcare systems are based on legacy and inherit “the way it has always been done” to avoid risks. But this legacy does not always align with the changing needs of the system and its actors. For instance, there seems to be a predicament of matching the traditional healthcare research to policy lifecycle to the speed of commercial technology innovation and changing digital health needs (39). In the traditional method, innovations will undergo extensive testing to prove safety and efficiency, taking up to six years (in the USA). By the time testing and evaluation has been completed, hundreds of new technological innovations will have flooded the market, current systems and infrastructures will have changed or upgraded, and even the needs of the end-users will have changed considerably with the knowledge of innovation advancements. This mismatch results in many DHI decisions being made without the guidance and security that has always been provided, leaving decision makers with legal and ethical concerns regarding the matter. Risk adverse organizations, such as in healthcare, may shy away from these decisions.

The implementation barrier of challenges with infrastructure has much to do with the tension between changing needs and dependency on past decisions, resource scarcity, and institutionalism in healthcare. Past decisions determine whether the current infrastructure can support DHI implementation. Institutionalism and its multi-layer decision-making processes together with resource scarcity create substantial obstacles to adapting, changing, or upgrading such infrastructures. Both Nilashi 2016 and Greenhalgh 2019 have incorporated infrastructure challenges in their TMFs, arguing that new DHIs need to be compatible with the existing infrastructures. Greenhalgh further showed how large commercial non-health digital innovations may evolve to become incompatible because they lack the understanding that health infrastructures do not adapt easily when technology is updated.

Another notable paradox we found is the contrast between the need for social and professional interdependence (across organizations) with the emerged differences in organizational strategies, priorities, and practices as well as individual health provider autonomy. Variances in procedures and practices are a result of autonomy but are also often embedded in organizational policies, rules, and regulations, making them difficult to adapt. DHIs are supposed to support patient-centered care (7) but can only do so when professionals and organizations are open to participate in collective action under common leadership. The differences in individual and organizational practices are particular pain points for technology developers because it makes fitting the DHI to the organization and end-user needs extremely difficult, which can be a cause of poor fit of the technology.

Healthcare systems are resource strained and face several other barriers to implementing digitalization, such as the need for external and/or additional expertise and fiscal, cognitive, and time investments. Nilashi 2019 and Deng 2021 both incorporated the financial risk of DHI implementations in their TMFs as a significant consideration. Involvement of external expertise relates to increased costs associated with implementation. Nilashi 2019 acknowledge that organizations would consider it a barrier if their inhouse ICT department does not possess the necessary skills and expertise for the DHI implementation. Literature on inequalities in digital health interventions cite the lack of resources, including involvement of external expertise and technical support, as a major barrier to health digitalization (40, 41).

Resource strain hinders the financial, time, and cognitive investments required for DHI implementation, learning, and adapting to new ways of working. This opposition explains the major barriers to DHI adoption and acceptance of time- and workload-related concerns and lack of training and education. Constructs such as “perceived ease of use” in the end-user behavior suggest that, when DHI implementation require a high learning investment, it reduces intention to (re)use the DHI. In response, Boussadi 2011 suggested that health digitalization processes should start with simple digital functions which have a low investment before attempting to scale up to highly advance DHI functionalities. The study by Byrd 2021, however, shows us that once the DHI use is well established in a certain work environment, perceived critical mass helps to overcome the barrier effects of perceived ease of use.

Finally, we also identify a tension between resource strain and healthcare as a commodity. Market logic in healthcare demands an increase in the amount and quality of services offered, which inherently implies higher costs. Although DHIs are meant to be an affordable solution to the demands, they still require a significant investment. It is for this reason that Nilashi 2016 and Deng 2021 have included the constructs “relative advantage” and “price rationality” in their TMFs. They both argue that the demand and benefits of such services need to outweigh the costs. Unfortunately, the benefits of DHIs are often measured in fiscal income or reduction in costs and not in terms of quality of care. Consequently, the risk arises that e-health services become a luxury for those that can afford to take risks rather than the norm for those who need the efficiency and empowerment DHIs can provide. Academic literature on the topic of digital health interventions agree that major inequalities are caused or sustained by digital health services rather than being alleviated with reduced DHI adoption by population groups with lower socio-economic-status, in rural and remote areas, or living with disabilities (42, 43). The main reason for inequality in health digitalization is the lack of financing for hardware, infrastructure, and training. Nilashi 2016 further elaborates that larger organizations in more well-off areas simply have more funding to support DHIs.

One major reason for the failure to sustain continuous use is that DHI's are considered disruptive innovations, meaning that the adoption of these technologies require a profound shift in working culture, organization of work, and practices (5, 7, 14–17). These changes need to occur within the unique environment of the healthcare system, which is both rigid and fluid. The key may be to navigate all of these different perspectives and deal with the tensions between them in order to incorporate DHIs successfully in practice.

### 4.2 Strengths and limitations

To our knowledge, this is the first review to analyze and synthesize the authors' reasoning for inclusion of different constructs to identify perspectives or assumptions about the healthcare system that play a pivotal role in health digitalization.

This review has some limitations. Our selection criteria focused specifically on health digitalization, including only studies with empirical datasets, DHIs involving commercial stakeholders, and innovations that demonstrate the disruptive impact of digital transformation. Commercial parties were considered external actors who offer IT products or services to healthcare organizations or practitioners in exchange for payment. We made deliberate methodological choices in terms of the search terms we used to reflect our intended scope and focus. However, focusing on specific terminologies such as “digitalization” and “digital transformation” carry an inherent risk of excluding broader TMFs with partially overlapping or complementary insights. One such framework that was not included in our search results and subsequently identified during the review process is the Non-adoption, Abandonment, Scale-up, Spread, and Sustainability (NASSS) framework by Greenhalgh et al. (20). This example reflects the diversity of language and terminology use in the field, thereby emphasizing the vastness and multidisciplinary nature of healthcare digitalization. It also highlights the value of peer-review processes and collaboration across disciplines in comprehensively covering and interpreting the literature. We do not claim that this review is all-inclusive of all contextual factors in health digitalization, but it does give an overview of empirically supported digitalization-specific TMFs and their related factors and constructs. It is possible that within the wider literature regarding DHI implementation, and using broader search terms, more considerations and recommendations will come to light.

We further acknowledge that the heterogeneity of the included TMFs, constructs, and factors complicate synthesis. Even though we worked hard to carefully and comprehensively analyze each article, TMF, and its components, there may be some variances and discrepancies in the analysis and results. For instance, the three groups we identified are not based on previous scientific work but on our observation of the data. The richness and complexity of the data created a challenge for categorizing the TMFs. In some of the studies, such as Harvey 2013, it was unclear which category best fit the TMF and the final allocation was based on discussions among the research team to determine the most appropriate group.

The heterogeneity of the data also means that synthesis and conclusions are rather broad and overarching and do not depict clear and concise actions for different actors. The review does provide the reader with a good oversight of some of the most important healthcare system characteristics that shape health digitalization processes, facilitators, and barriers, which is invaluable to preparing health digitalization strategies. The oversight is rooted in multiple perspectives from both inside and outside of healthcare, facilitating a rich understanding of the field.

We would like to highlight that the included TMFs were mostly based on empirical evidence from public healthcare systems with a fixed salary approach. More research is needed to explore health digitalization logics and the influence of contextual factors in healthcare systems with a more privatized or fee-for-service structure.

### 4.3 Recommendations for future research

We gave an oversight of empirically tested TMFs and how they are located within the multi-dimensional field of innovations, actors, and digital landscapes. We have highlighted the differences between TMFs with different end-goals and implied actor-groups. However, more research is needed into the roles, functions, and traits of specific actors in this ecosystem and how they establish trust, stability, and collaboration within a dynamic system.

## 5 Conclusion

Implementation of DHIs remains challenging, with high rates of failure and non-adoption. A heterogeneous array of theories, models, and frameworks (TMFs) exist to explain, predict, or guide the DHI lifecycle from design and development to implementation and dissemination. These TMFs are specific to their purpose, context, type of innovation, and intended scope of implementation Understanding these differences is of great importance to support their appropriate use.

This review contributes to the field by offering a more structured understanding of empirically based TMFs for health digitalization, supporting their appropriate use. We outlined a landscape of healthcare systems from multiple perspectives relevant to health digitalization and consolidated related recommendations. Together, the findings present a solid foundation that can support health digitalization strategies and projects as well as the appropriate selection of TMFs in different contexts and stages of the innovation lifecycle.

## Data Availability

A summary of original contributions presented in the study are included in the article/Supplementary material. All original research articles, except for Boussadi, 2011 are available via open access. Further enquiries can be directed to the corresponding author.
