# Supplementary material for: How do domain diversity and healthcare system assumptions shape implementation research? A systematic review of theories, models, and frameworks for health digitalization
Source: Front Digit Health. 2026 Jul 16;8:1812300. doi: 10.3389/fdgth.2026.1812300 (PMC13422491; doi:10.3389/fdgth.2026.1812300)
Supplement: Supplementary file 1 [file Datasheet1.pdf]

Supplement A: Search strategy

PubMed (1757)

Search	Query	Results
#4	Search: **#1 AND #2 AND #3**	1,757
#3	Search: **"Stakeholder Participation"[Mesh] OR Actor*[tiab] OR Promotor*[tiab] OR "Cross organizational*"[tiab] OR Champion*[tiab] OR "Multi stakeholder*"[tiab] OR Provider*[tiab] OR Business*[tiab] OR organization[tiab] OR organisation[tiab] OR "living Lab*"[tiab]**	760,197
#2	Search: **"Digital Technology/instrumentation"[Mesh] OR "Digital Technology/organization and administration"[Mesh] OR "Digital Technology/trends"[Mesh] OR "tool*"[tiab] OR "platform*"[tiab] OR "software"[tiab] OR "digital transformation"[tiab] OR "digital maturit*"[tiab] OR "digitalization*"[tiab] OR "digitization*"[tiab] OR "telecommunication"[tiab] OR "digital collaboration"[tiab] OR "digital eco-system*"[tiab]**	1,626,139
#1	Search: **"Models, Educational"[Mesh] OR "Models, Organizational"[Mesh] OR "Transtheoretical Model"[Mesh] OR "Models, Theoretical"[Mesh:NoExp] OR "theoretical model*"[tiab] OR "theoretical framework*"[tiab] OR "theoretical pathway*"[tiab] OR "theoretical strateg*"[tiab] OR "theoretical approach*"[tiab] OR "theoretical test*"[tiab] OR "theoretical step*"[tiab] OR "theoretical trajector*"[tiab] OR "theoretical mechanism*"[tiab] OR "theoretical action*"[tiab] OR "theoretical collective action*"[tiab] OR "theoretical foster*"[tiab] OR "theoretical tool*"[tiab] OR "theoretical governance*"[tiab] OR "theoretical role*"[tiab] OR "theoretical interaction*"[tiab] OR "theoretical process*"[tiab] OR "theoretical change management*"[tiab] OR "framework approach"[tiab]**	256,917

Embase (1195)

Search	Query	Results
#4	Search: #1 AND #2 AND #3	1195
#3	'stakeholder participation'/exp OR 'actor*':ti,ab,kw OR 'promotor*':ti,ab,kw OR 'cross organizational*':ti,ab,kw OR 'champion*':ti,ab,kw OR 'multi stakeholder*':ti,ab,kw OR 'provider*':ti,ab,kw OR 'business*':ti,ab,kw OR 'organization':ti,ab,kw OR 'organisation':ti,ab,kw OR 'living lab*':ti,ab,kw	1,134,484
#2	('digital technology'/exp AND 'devices'/de) OR ('digital technology'/exp AND 'organization and management'/exp) OR ('digital technology'/exp AND 'trend$':ab,ti,kw) OR 'tool*':ti,ab,kw OR 'platform*':ti,ab,kw OR 'software':ti,ab,kw OR 'digital transformation':ti,ab,kw OR 'digital maturit*':ti,ab,kw OR 'digitalization*':ti,ab,kw OR 'digitization*':ti,ab,kw OR 'telecommunication':ti,ab,kw OR 'digital collaboration':ti,ab,kw OR 'digital eco-system*':ti,ab,kw	2,200,923
#1	'educational model'/exp OR 'transtheoretical model'/exp OR 'theoretical model'/de OR 'theoretical model*':ti,ab,kw OR 'theoretical framework*':ti,ab,kw OR 'theoretical pathway*':ti,ab,kw OR 'theoretical strateg*':ti,ab,kw OR 'theoretical approach*':ti,ab,kw OR 'theoretical test*':ti,ab,kw OR 'theoretical step*':ti,ab,kw OR 'theoretical trajector*':ti,ab,kw OR 'theoretical mechanism*':ti,ab,kw OR 'theoretical action*':ti,ab,kw OR 'theoretical collective action*':ti,ab,kw OR 'theoretical foster*':ti,ab,kw OR 'theoretical tool*':ti,ab,kw OR 'theoretical governance*':ti,ab,kw OR 'theoretical role*':ti,ab,kw OR 'theoretical interaction*':ti,ab,kw OR 'theoretical process*':ti,ab,kw OR 'theoretical change management*':ti,ab,kw OR 'framework approach':ti,ab,kw	165,064

Web of Science (3003)

Search	Query	Results
#4	Search: #1 AND #2 AND #3	3003
#3	TS=(“actor*” OR “promotor*” OR “cross organizational*” OR “champion*” OR “multi stakeholder*” OR “provider*” OR “business*” OR “organization” OR “organisation” OR “living lab*”)	1,699,687
#2	TS=(“tool*” OR “platform*” OR “software” OR “digital transformation” OR “digital maturit*” OR “digitalization*” OR “digitization*” OR “telecommunication” OR “digital collaboration” OR “digital eco-system*”)	3,362,723
#1	TS=(“theoretical model*” OR “theoretical framework*” OR “theoretical pathway*” OR “theoretical strateg*” OR “theoretical approach*” OR “theoretical test*” OR “theoretical step*” OR “theoretical trajector*” OR “theoretical mechanism*” OR “theoretical action*” OR “theoretical collective action*” OR “theoretical foster*” OR “theoretical tool*” OR “theoretical governance*” OR “theoretical role*” OR “theoretical interaction*” OR “theoretical process*” OR “theoretical change management*” OR “framework approach”)	259,326
