# Supplementary material for: How do domain diversity and healthcare system assumptions shape implementation research? A systematic review of theories, models, and frameworks for health digitalization
Source: Front Digit Health. 2026 Jul 16;8:1812300. doi: 10.3389/fdgth.2026.1812300 (PMC13422491; doi:10.3389/fdgth.2026.1812300)
Supplement: Supplementary file 2 [file Datasheet2.pdf]

TMFs DEALING WITH DESIGN AND DEVELOPMENT OF DHI			
	**Boussadi, 2011**	**Nikayin, 2013**	**Pietronudo, 2022**
**Adequacy**	Describes and tests a method (based on business process modelling and unified processing) to identify and design the algorithms (business rules) for a pharmaceutical validation and alert system.<br>Method was comprehensively described including all steps, artifacts, actors and construct components.	The theory attempts to validate some relations between preselected constructs from a literature review, but does not address environmental or industry factors to predict behaviour.	The theory is moderately adequate due to its somewhat limited scope and lack of including forces from organizational factors.
**Clarity**	The theory is mostly clear of the main components to be considered and also offers a table with artifacts to be obtained from the individual steps. However the theory is not always clear to follow (at first read) what is meant by each component.	All constructs clearly defined and described. Relations between constructs clearly identified	Relatively clear but not all constructs are clearly defined and the figure of the model is complicated to follow. Some constructs are described in the text but then grouped together in the final figure.
**Consistency**	Good consistency of terminology throughout the model and model description	Good consistency of terminology.	Some discrepancies of terms used making the model somewhat difficult to follow.
**Logical development**	Follows on previous work in systems engineering that is well documented, but only illustrated by the means of one real life and one documented case study.	Sub-optimal methodological practices were used to support and validate the theory. Constructs were preselected from the literature. The use of a single case study to support results reduces generalizability or commercial use of the theory. Also, the authors relied on inferred code networks to determine relations between constructs, which might be influenced by the authors' subjective interpretation.	The hypothesized theory is based on an extensive review of previous work. The single case study design (based on interviews) to illustrate the theory may reduce the reproducibility of the theory.
**Level of theory / abstraction**	Model more on practice level with concrete steps to be followed during the process.	Medium high level of abstraction with concepts addressing complex themes (such as leadership) and not so much specific factors.	Medium high level of abstraction with mostly transferable constructs. The description of the constructs within the context contains is more specific with concrete examples.
**Complexity**	Fairly complex with main constructs (process steps), related artifacts and incorporation of sub-systems and classifications.	The theory as such has low complexity, but does not address the complexity of its individual constructs.	The theory is moderately complex with different levels of constructs interacting.

**Discrimination**	Unique. Applying principles from systems engineering to the design and development of a pharmaceutical validation tool	The theory is unique, evolving from collective action theory and business ecosystem theories. Constructs within the theory does not occur in other theories included in this systematic review.	The theory provides unique perspective drawing from work done in the domains of management and economic sciences and applying it to the healthcare industry.
**Reality convergence**	Underlying assumptions are true and correct, although they are from the domain of business studies.	Underlying assumptions are based on previous scientific papers.	Underlying assumptions are based on previous scientific research. Assumptions can not be disproved by the literature.
**Pragmatic**	The theory can be operationalized in practice. It is a process that is clearly described, as well as concrete documentation that will result from the implementation of the theory.	The theory can be operationalized by means of good governance and leadership. However, the theory is context dependent and specific environmental factors (such as government support, for-profit vs not-for-profit organizations) may influence the reliability of the theory.	The theory is pragmatic with concrete and specific empirical implications for platform providers in the healthcare sector.
**Scope**	The theory is however narrow and specific, focusing on a systematic process to identify specific abstract elements of the clinical support system implemented. The theory thus focuses on one part of the design phase within the innovation lifecycle with less regard for dissemination and improving behavioural intention of end-users.	The theory has broad scope, universal constructs, but the outcomes, and relations between the constructs might be more context dependent.	Medium narrow scope of constructs that is being widened in the description of the constructs. The focus lies on the capabilities and roles of platform intermediaries (within the healthcare context) but does not extend to how these capabilities impact healthcare organizations.
**Significance**	The theory can have a significant impact on how the unified modelling language can be used, within the health care sector, to ensure the accurateness of DHI content and consequently its performance. This method could also be deployed in the development of standards for DHI's.	The theory can be used to guide leadership and partnership selection among multi-organizational collaborative efforts in DHI development. Other constructs are non-adaptive and can only describe a phenomena, rather than significantly influence it.	Highly significant research but should be explored in a broader context.
**Utility**	Further studies should still be done to assess the impact of using this process on the DHI's interoperability, perceived usefulness, acceptance and other clinical outcomes	The theory could generate further explorative research questions in the domain of selective incentives and governance styles that are beneficial to DHI development and dissemination.	Good utility enabling many new working hypothesis mainly for digital platform developers.

TMFs dealing with organizational adoption of DHI			
	**Harvey et al. (2012)**	**Nilashi et al. (2016)**	**Greenhalgh et al. (2019)**
**Adequacy**	The theory comprehensively addresses the work organization (way of working) of pharmacies with a high level of technology acceptance and usage as opposed to pharmacies with a low level of technology acceptance and innovation. The theory is fairly adequate in its scope but does not address work culture, work hierarchical relations, layout of special resources and accessibility of technological devices.	The theory is adequate in its scope by combining constructs from multiple other theories and models into a consolidated hierarchy including factors significant in the healthcare industry such as security concerns.	The theory is adequate in its scope of infrastructure determinants and their effect on DHI adoption and implementation but lack vendor involvement.
**Clarity**	The theory has good clarity and consistent, easy to understand terminology.	All constructs are clearly defined and described.	All constructs are clearly defined and described.
**Consistency**	Good consistency of terminology and constructs.	Good consistency of terminology.	Good consistent use of terminology.
**Logical development**	Based on results of an extensive literature review both in the domain of socio-technical sciences as well as in the domain of pharmacy practice. The framework was then populated with comparative results from extensive multi-site ethnographical observations and interviews. However it not entirely clear how the authors defined a techno-centred pharmacy.	The theoretical framework of the model is grounded in a thorough literature review of the most common used and researched models. Validated by means of appropriate statistics (fuzzy network analytic process).<br>Sample size was relatively small (n=20) and did not include top-management. This means that responses may not include many regulatory, medicolegal, business and financial concerns. Answers from different groups of stakeholders, with vastly different interests were combined, meaning that the overall results may rather reflect the heterogeneity of interests than actual construct importance.	The theory is based on a case study which is generally argued to be somewhat limiting in substantiating and generalizing findings. However, the theory is well grounded on the work of previous scientific research and substantiated using a meticulous and exhaustive ethnographic study.
**Level of theory development / abstraction**	Model is on a more practical level and less abstract.	Medium level of abstraction with somewhat generalized constructs from previous non-specific DHI implementation theories and models, combined with more specific constructs.	Medium-low level of abstraction with mostly concrete non-abstract constructs specific to infrastructure. One construct (the construct of embeddedness, relational and emergent and the construct) is somewhat more abstract than

			the others, pertaining to the philosophical ideas of existence.
**Complexity**	Relatively uncomplex with main constructs and three different types of pharmacies are then compared to one another within each construct.	The theory is low complexity composed of two levels: the factors and subfactors.	The theory has somewhat complex constructs but simple relations between the constructs. The constructs are described to have many influential factors that may shape them.
**Discrimination**	Unique perspective looking at work organizational and occupational factors that were not found in any of the other theories.	The theory derives its factors from other theories in the research area. It is unique in that it provides a hierarchical relationship to the collective set of factors from other most used theories.	The theory is discriminatory from other theories in this systematic review with a very unique perspective on DHI adoption and implementation incorporating the role of dynamic and static infrastructure qualities.
**Reality convergence**	Assumption of the real world seem to be realistic and stemming from cited scientific papers.	Underlying assumptions are true for the study setting. Contextual factors such as healthcare financing, organizational policies and culture may all influence the outcome of the study, but were not discussed.	Underlying assumptions are based on previous scientific work.
**Pragmatic**	The theory is moderately pragmatic with clear work organization conditions of pharmacies who are technology orientated. However it is not clear whether the work organization is a precursor to as a result of technology adoption and implementation.	The theory is descriptive and slightly abstract and therefore might have lower pragmatism.	The theory is fairly pragmatic but needs many additional resources from a variety of actors to be operationalized which makes it complicated in an institutionalized environment as proven by the case study.
**Scope**	The theory is somewhat specific in scope, describing the work organization of a pharmacy, but does not link it other contextual factors and relations.	The theory has broad scope, universal constructs, but the outcomes, and relations between the constructs might be more context dependent.	Broad scope on the topic of infrastructure for DHI adoption and dissemination within a hospital group (four settings) but does not include vendor involvement or how the constructs are realized in a primary healthcare setting.
**Significance**	The theory can be significant but will require resources and appropriate management to implement in the real world.	The theory addresses important factors in the domain of hospital adoption of DHI which is a crucial step in DHI implementation.	The study is of high significance focusing on often overlooked critical determinants of health technology.
**Utility**	The theory is able to generate further hypothesis to be tested, in specific the relation between contextual, environmental and organizational factors and work orientation.	The theory can give a framework for developers to guide product development according to top-management considerations. The theory should however undergo further testing and validation.	The theory has high utility value being able to generate numerous new hypothesis and potential improvements and considerations to infrastructure for DHI adoption and implementation.

TMFs dealing with organizational adoption of DHI continue	
	**Deng et al. (2021)**
**Adequacy**	The theory incorporates the three most used technology acceptance models to give a broad, although not very specific, overview of factors influencing individual technology adoption behaviour. The theory should be supplemented with other significant theories to illuminate details in the organizational and industrial landscape that shape these agent-centred constructs.
**Clarity**	Clear description of constructs and subconstructs.
**Consistency**	Consistent use of terminology
**Logical development**	Theory is an extension of previous scientific work. Unfortunately it is not clear how the authors decided to include certain constructs in the new consolidated theory, while omitting others. The new theory was validated using SEM and explorative factor analysis on data retrieved from an acceptable sample size that included some randomization to reduce bias. However a significant majority of respondents were male, and the authors did not disclose how the survey was distributed (e.g. by post or electronic means) which may mask the inherent possibility if bias.
**Level of theory / abstraction**	Moderately high level of theory abstraction with some more generic and encompassing constructs that are dependent on other contextual factors such as technology sharing willingness and technology absorptive willingness.
**Complexity**	Low complexity and easy to understand.

**Discrimination**	The theory is derived from combining selected constructs from three foundation theories namely TAM, TOE and DOI. The theory is discriminatory to the extent that it is a consolidated theory from known constructs and the development of two new organizational constructs namely technology absorptive willingness and technology sharing willingness.
**Reality convergence**	Assumptions are grounded in previous scientific research.
**Pragmatic**	The theory is relatively pragmatic to understand and measure factors influencing DHI adoption and diffusion but due to the level of abstraction it is less clear how to use the theory for the purpose of improving DHI adoption and diffusion.
**Scope**	The theory has a broad scope covering agent-centred, organizational and industrial constructs related to the topic.
**Significance**	The theory addresses some important constructs over a wide scope that influences DHI adoption and dissemination and its significance lies therein that it provides a single measurement tool to determine influencing factors from a variety of foundational theories.
**Utility**	The theory can in general give direction to new hypothesis, but will most likely need some additional information in order to generate a specific detailed working hypothesis for intervention.

TMFs dealing with behavioural change of end-users			
	**Beglaryan, 2017**	**Yousef, 2022**	**Jin et al. (2024)**
**Adequacy**	Generally the theory is adequate for its goal, which was to explore constructs related to physicians' acceptance of an EHR system. The theory considers both individual constructs (derived from the TAM) as well as organizational level constructs (derived from neo-institutional theory and multi-level framework of technology adoption (MFTA)).	The theory focused on overarching themes but does not adequately address (or necessarily consider) the nuanced complexities that make up the different themes. According to the authors, the theory explains 73% of the variance seen in user acceptance. The theory also had limited inclusion of themes as factors specific to primary health care.	The theory is adequate in its objective to explore the relation between perceived service quality, electronic word-of-mouth (e-WOM), satisfaction and behavioural intention. However the scope is limited addressing only a small section of the technology adoption and diffusion landscape.
**Clarity**	Constructs and their relations are clearly defined.	Constructs of the theory are clear and well defined, but lacks some clarity on the factors that form the basis of each overarching construct.	Constructs and relations are clearly defined.
**Consistency**	Consistent use of terminology.	Some terminology was not always used in a consistent fashion with related, but different, semantics used for the same construct such as behavioural intention to support the technology, behavioural intention to use the technology and acceptance of the technology that seemed to be used interchangeably.	Consistent use of terminology
**Logical development**	The study follows a logical development and extension of the well known Technology Acceptance Model (TAM), with added constructs from neo-institutional theory and MFTA.	The study follows as a logical extension of previously published research. The study consisted of a survey, but the authors cannot determine the response rate of the survey. Because this was an online questionnaire, the study might have been prone to response and availability bias. The specific questionnaire was not validated, but only pilot tested. It was however developed from adapting several other questionnaires from studies testing technology acceptance factors, but the number of items was drastically reduced from 63 to 16 shedding doubt on the validity of the questionnaire. Some questions in the	The theory and hypothesis are logically developed from previous research relevant to the specific setting. SEM analysis was done on a large sample (n=593) with good distribution of gender, age, education level and monthly income. The survey used questions from previous validated measurement tools, and the new combined measurement tool has been assessed for validity and reliability. Online surveys however have an inherent availability and self-selection bias.

		questionnaire were non-specific to the construct intended to be tested.	
**Level of theory / abstraction**	Low to moderate level of abstraction. Most of the constructs are concrete and simple. However there are three constructs, namely 'patient influence', 'resistance to change' and 'organizational change' that are of a more higher level of abstraction with underlying mechanisms and sub-constructs.	The theory constructs are well abstracted into more general themes with no specific subthemes defined.	Moderate high level of abstraction with somewhat generic constructs which each have several influencing factors.
**Complexity**	Low complexity and easy to understand.	Low complexity with easy to understand constructs and relations.	Low complexity.
**Discrimination**	The study is unique in combining known TAM constructs with organizational (meso) level constructs specifically applied to physicians' acceptance of the DHI.	The study mainly repeated the results from other UTAUT studies, but in a new setting. The study did also test the influence of age, professional role and experience on the construct relations within the UTAUT theory, but found no correlations, meaning that the theory is still similar to UTAUT, but the study is somewhat more discriminatory.	The theory is an extension of the theory of planned behaviour with the addition of electronic word of mouth (e-WOM) and perceived service quality borrowed from consumer science research. The perceived service quality could be seen as affiliated constructs to and elaborative of perceived ease of use and perceived usefulness by also including vendor support in the form of interaction quality. The inclusion of e-WOM is unique to this study.
**Reality convergence**	Assumptions were grounded in previous scientific literature. The authors did (as in many other studies) use behavioural intention as a proxy for testing behavioural adoption, and although the two constructs are related, they won't necessarily have the same result.	Assumptions were grounded in previous scientific literature. The authors did (as in many other studies) use behavioural intention as a proxy for testing behavioural adoption, and although the two constructs are related, they won't have the same result.	Underlying assumptions grounded in scientific research.
**Pragmatic**	Fairly pragmatic, but the more abstracted constructs might need further breakdown and deepening in order to truly grasp the context.	The high level of abstraction without defined subfactors of each construct reduces the pragmatism of the theory.	The authors used the theory to substantiate some practical empirical suggestions for continuous adoption behaviour improvements. These suggestions are mainly directed towards platform vendors focusing on quality assurance and promotion of benefits.
**Scope**	Moderate scope stretching over the individual (micro) and organizational (meso) level. The authors took care as to only focus on constructs	The scope addresses a wide variety of themes, but still lack some of the themes specific to medical informatics such as privacy and	The theory has a limited scope focusing on some actor-centred constructs that are influential to behavioural intention but does

	relevant to physicians' attitudes and behavioural intention and not broader organizational factors that influence organizational adoption.	security, vendor roles and responsibilities and interoperability with other systems for instance.	not elaborate on organizational, industrial and societal determinants.
**Significance**	The constructs tested are all relevant to physicians' acceptance of the technology.	The themes tested are relevant to the topic of technology acceptance and adoption.	The theory is partly significant in exploring the relation of e-WOM to other technology adoption factors as e-WOM is a significant player in predicting and influencing consumer behaviours.
**Utility**	The model can drive further hypothesis and even advise implementation strategies. The focus of this study was physicians, but DHI acceptance (as a whole) often include other disciplines within the healthcare system as well. Therefore further testing of the model in an expanded population group will be needed.	The theory can drive further hypothesis strategies that can be used to promote technology acceptance and diffusion. However in many cases some additional information will be needed to generate specific strategies due to the high level of abstraction of the theory constructs.	The theory can guide new hypothesis but would have to be combined with knowledge of contextual determinants and how this theory relates to other technology adoption theories. The relation between e-WOM and personal constructs such as attitudes, subjective norms and perceived critical mass should be further explored.

TMFs dealing with behavioural change of end-users continue		
	**Byrd et al. (2021)**	**Zhang et al. (2024)**
**Adequacy**	The theory is fairly adequate for its intended purpose, which is to determine the relation between perceived critical mass and TAM constructs. However perceived critical mass consists of observability, demonstrability and conversations about the technology that were not individually included. Other constructs were also excluded such as social influence on the assumption that critical mass will replace social influence as a more relevant (but affiliated) construct.	Theory and associated factors are comprehensively described. The authors also explains how this theory is related to other theories developed for health technology implementation and adoption.
**Clarity**	Constructs, hypothesis and relations of constructs were clearly defined in the study.	Main constructs are clearly defined and explained.
**Consistency**	Terms are used consistently and the authors differentiate clearly between similar, but not identical terms.	Consistent use of terminology throughout the report.
**Logical development**	The theory is a logical extension of the TAM by adding critical mass and personal innovativeness in technology (borrowed from DOI theory) as constructs to make a new theory. While the underlying assumptions may be logical, they have not always been scientifically proven.	Logical development of hypothesis, grounded in previous scientific research and well established theories. The hypotheses were tested using SEM analysis of online survey responses. Although the sample size was fairly large the use of online surveys may inherently expose the study to availability and self-selection bias.<br>The measurement tool was relevant and statistically tested for validity and reliability.
**Level of theory / abstraction**	Fairly low level of abstraction with concrete constructs.	Moderately high level of abstraction in the theory, but related lower level constructs were explained in the discussion.
**Complexity**	Low complexity.	Moderate level of complexity as the constructs of network externalities are complex constructs with many influencing factors. The relations between network externalities, confirmation, perceived value, satisfaction and continuous adoption behaviour is easy to understand.
**Discrimination**	The theory does stem from two other well known theories, but is differentiated in that the relations between critical mass and other constructs are statistically supported.	The theory does overlap with previously well researched theories (expectation confirmation model and technology acceptance model) with the discriminatory addition of network externalities. Network externalities can be compared to the affiliated construct of perceived critical mass and social influence from the Dissemination of

		Innovation model and the study of Byrd et al. (2021) as the network externality theory also addresses the “chicken and egg” problem commonly seen in platform implementation and dissemination. The network externality theory appears to be more nuanced and specific in the way they relate to other constructs specifically focusing on system value, and benefits to parties involved.
**Reality convergence**	The authors assumed critical mass will be more important than social influence and that CM will have an additional effect through the effect of social influences. Although logic, these assumptions are not empirically validated.	Underlying assumptions grounded in scientific research in the domain of psychology and consumer sciences.
**Pragmatic**	The theory is pragmatic providing the reader with clear and grounded information to adjust strategies according to the current level of dissemination of a digital health innovation. Because the theory does not address other aspects of technology acceptance such as integration with the workflow, overcoming resistance and other organizational and environmental factors, the	Due to its level of abstraction the theory does not provide concrete steps to increase platform value directly but provides guidance towards an area to consider when developing and implementing healthcare platforms.
**Scope**	The scope of the theory is fairly limited and does not address other organizational and environmental factors that may influence perceived critical mass, perceived usefulness, perceived ease of use and behavioural intent.	Moderately broad scope including patients and professionals. The constructs of network externalities encompass a wide range of factors that influence perceived value.
**Significance**	The construct of critical mass is known to be important in the domain of digital platform acceptance and adaptation as it has been shown to be relevant in overcoming the “chicken and egg” problem of low usefulness due to low usage that is commonly seen with digital platforms.	Theory has high significance as it addresses platform continuous adoption challenges from the perspective of network externalities and network value. This is a novel approach that offers additional insight solving health platform related challenges while still
**Utility**	The theory can drive further hypothesis and creative ideas on how to increase perceived critical mass. Some lower level abstracted concepts might still be needed to support the development of refined implementation strategies.	The theory can be used to generate new hypothesis in the field of value creation for platform users.
